# Increased expression of miR142 and miR155 in glial and immune cells after traumatic brain injury may contribute to neuroinflammation via astrocyte activation

**DOI:** 10.1111/bpa.12865

**Published:** 2020-06-26

**Authors:** Anatoly Korotkov, Noora Puhakka, Shalini Das Gupta, Niina Vuokila, Diede W. M. Broekaart, Jasper J. Anink, Mette Heiskanen, Jenni Karttunen, Jackelien van Scheppingen, Inge Huitinga, James D. Mills, Erwin A. van Vliet, Asla Pitkänen, Eleonora Aronica

**Affiliations:** ^1^ Department of (Neuro)Pathology, Amsterdam Neuroscience Amsterdam UMC, University of Amsterdam Meibergdreef 9 Amsterdam 1105 AZ the Netherlands; ^2^ Department of Neurology, A. I. Virtanen Institute for Molecular Sciences University of Eastern Finland Kuopio FI‐70211 Finland; ^3^ Department of Neuroimmunology Netherlands Institute for Neuroscience Meibergdreef 47 Amsterdam 1105 BA the Netherlands; ^4^ Swammerdam Institute for Life Sciences, Center for Neuroscience University of Amsterdam Science Park 904 Amsterdam 1090 GE the Netherlands; ^5^ Stichting Epilepsie Instellingen Nederland (SEIN) Heemstede the Netherlands

**Keywords:** biomarker, gliosis, inflammation, microRNA, secondary injury, TBI

## Abstract

Traumatic brain injury (TBI) is associated with the pathological activation of immune‐competent cells in the brain, such as astrocytes, microglia and infiltrating immune blood cells, resulting in chronic inflammation and gliosis. This may contribute to the secondary injury after TBI, thus understanding of these processes is crucial for the development of effective treatments of post‐traumatic pathologies. MicroRNAs (miRNAs, miRs) are small noncoding RNAs, functioning as posttranscriptional regulators of gene expression. The increased expression of inflammation‐associated microRNAs miR155 and miR142 has been reported after TBI in rats. However, expression of these miRNAs in the human brain post‐TBI is not studied and their functions are not well understood. Moreover, circulating miR155 and miR142 are candidate biomarkers. Therefore, we characterized miR142 and miR155 expression in the perilesional cortex and plasma of rats that underwent lateral fluid‐percussion injury, a model for TBI and in the human perilesional cortex post‐TBI. We demonstrated higher miR155 and miR142 expression in the perilesional cortex of rats 2 weeks post‐TBI. In plasma, miR155 was associated with proteins and miR142 with extracellular vesicles, however their expression did not change. In the human perilesional cortex miR155 was most prominently expressed by activated astrocytes, whereas miR142 was expressed predominantly by microglia, macrophages and lymphocytes. Pro‐inflammatory medium from macrophage‐like cells stimulated miR155 expression in astrocytes and overexpression of miR142 in these cells further potentiated a pro‐inflammatory state of activated astrocytes. We conclude that miR155 and miR142 promote brain inflammation via astrocyte activation and may be involved in the secondary brain injury after TBI.

## Introduction

Traumatic brain injury (TBI) is a major cause of death and disability in humans, which is estimated to affect more than 50 million people worldwide every year (12, 32). It involves a number of pathological alterations as a result of external damage to the brain (36, 40). Numerous molecular changes following the primary brain injury contribute to the secondary brain injury, which includes brain inflammation, reactive gliosis, blood‐brain barrier (BBB) dysfunction, axonal injury, progressive neuronal loss and remodeling of the extracellular matrix (ECM) (36, 41, 45, 53). Initial brain inflammation is mediated by immune‐competent cells, such as microglia and astrocytes, which produce pro‐inflammatory mediators, including cytokines interleukin 1 beta (IL‐1β) and tumor necrosis factor alpha (TNF‐α) (49, 63, 64). Sustained brain inflammation leads to chronic activation of glial cells and infiltration of neutrophils, lymphocytes and macrophages in the brain parenchyma (19, 49). This negatively affects the neuronal survival and promotes BBB dysfunction, further contributing to the secondary injury post‐TBI. A better understanding of the molecular and cellular alterations involved in brain inflammation is crucial for the development of effective and adequate management of post‐TBI neuropathology.

Brain inflammation can be regulated by microRNAs (miRNAs) (15, 50), which constitute a class of small noncoding RNAs, capable of posttranscriptional regulation of gene expression networks (7). MiRNAs in mammals suppress target gene expression by directing the RNA‐induced silencing complex toward the target messenger RNA (mRNA) through binding to the complementary regions in its 3′‐untranslated region. MiRNAs have been shown to regulate numerous biological processes within the central nervous system both under normal and pathological conditions (26). Transcriptomic studies have revealed many deregulated miRNAs in the cerebral cortex and hippocampus in animal models for TBI (13, 28, 43, 44, 61). Among them are two miRNAs that are crucially involved in the immune response and brain inflammation: miR155 (14) and miR142 (48). These miRNAs may participate in the secondary injury post‐TBI through the regulation of inflammation in the brain. Both these miRNAs have been found to be upregulated following the controlled cortical impact (CCI) injury in rats (16, 17, 38, 54, 62), but their expression in other post‐TBI models has not been characterized. Furthermore, the validation of these findings and the cell‐type specific characterization of miR155 and miR142 in the human post‐TBI brain are lacking. Finally, circulating miRNAs in blood have been hypothesized to reflect neuropathological changes occurring after brain injury (4, 13, 42). Interestingly, increased expression of miR142 was observed in serum 1 day after TBI in rats (6). Therefore, the potential of these miRNAs to serve as biomarkers deserves further investigation.

We hypothesized that the inflammation‐associated miR155 and miR142 are involved in the secondary brain injury and their expression in blood reflects the post‐TBI pathological processes in brain. Therefore, we investigated the expression and cellular localization of miR155 and miR142 in the perilesional cortex of rats after lateral fluid percussion injury (FPI) and in the human autoptic brain post‐TBI. The expression of miR155 and miR142 was analyzed in rat plasma post‐TBI. Additionally, the potential of miR155 and miR142 to modulate inflammatory response in human astrocytes was investigated in vitro.

## Materials and Methods

The detailed description of methods is available as Supporting Materials and Methods.

### Human brain tissue

The cases included in this study were obtained from the archives of the department of Neuropathology of the Amsterdam UMC, the Netherlands. Human postmortem brain samples (n = 3‐6, who died from TBI (Table 1), were included in this study, as well as five controls (n = 5) without a history of neurological diseases. The neuropathological evaluation of human TBI samples was performed by two trained neuropathologists and assessed according to a routine protocol. TBI was confirmed, which was associated with cerebral hemorrhage, gliosis and axonal injury in all TBI samples. The protocol for detecting axonal injury included luxol fast blue‐hematoxylin and eosin stainings, as well as immunohistochemistry for β‐APP and neurofilament proteins. Tissue was obtained and used in accordance with the Declaration of Helsinki and the Amsterdam UMC Research Code provided by the Medical Ethics Committee. All cases were reviewed by a trained neuropathologist. Brain tissue was fixed in 10% buffered formalin and embedded in paraffin.

**Table 1:** Clinical characteristics of patients.

Sample#	Gender	Age	Post‐TBI interval	Brain region	TBI cause
Control 1	F	27	–	Front	–
Control 2	M	60	–	Front	–
Control 3	F	35	–	Front	–
Control 4	M	31	–	Front	–
Control 5	M	67	–	Temp	–
TBI 6*	M	35	6 mon	Front	Horse kick
TBI 7	F	65	6 d	Front	Traffic accident
TBI 8	M	32	1 d	Temp‐par	Traffic accident
TBI 9	M	34	6 d	Front	Traffic accident
TBI 10	M	20	5 d	Front	Traffic accident
TBI 11†	M	67	1 mon	Front‐par	Traffic accident
TBI 12	M	52	38 y	Temp	Traffic accident

Post‐mortem human brain cortex of patients post‐TBI (n = 7) and post‐mortem control brain cortex (n = 5) samples were used in this study; F = female; M = male; TBI = traumatic brain injury, d = day(s); mon = months; y = years; Front = frontal cortex, Temp = temporal cortex; Par = parietal cortex.

* The case represented in Figure 2.

^†^ The case represented in Figure 5.

### Human primary cells

Primary fetal astrocyte‐enriched cell cultures were derived from human fetal brain tissue (14–20 weeks of gestation) obtained from medically induced abortions. All material was collected from donors from whom written informed consent for the use of the material for research purposes was obtained by the Bloemenhove clinic, the Netherlands. Tissue was obtained in accordance with the Declaration of Helsinki and the Amsterdam UMC Research Code provided by the Medical Ethics Committee. Human primary peripheral blood mononuclear cells (PBMCs) and T cells were isolated from blood using the Macs Pan T Cell Isolation Kit (Miltenyi Biotec, Bergisch Gladbach, Germany) and human primary microglia was isolated as previously described (35).

### Animals

Adult male Sprague Dawley rats (n = 20) (Envigo, Horst, the Netherlands) were housed in a controlled environment (temperature 22 ± 1 °C; humidity 50–60%; lights on from 07:00 to 19:00 h). At the time of the experiments the animals weighed 350–400 g. Water and pellet food were provided *ad libitum*. All animal procedures were approved by the Animal Ethics Committee of the Provincial Government of Southern Finland. All animal work was carried out in accordance with the guidelines of the European Community Council Directives 2010/63/EU.

### Lateral fluid‐percussion injury

Rats were randomly assigned to groups as follows: rats which received lateral FPI (n = 8), sham‐operated rats (n = 5) and naïve rats (n = 3). The rats were subjected to lateral FPI as described previously (22, 34). Brain injury was induced with a mean pressure of 3.18 ± 0.08 atm. Sham‐operated animals received anesthesia and underwent all surgical procedures without lateral FPI. The animals were sacrificed 2 weeks post‐TBI. Acute mortality within 48 h post‐TBI was 15% (3/20). One rat was excluded from the analysis because of a broken dura mater after TBI.

### Sample collection

Rats were anesthetized with 5% isoflurane and decapitated with a guillotine. The brain was dissected and the cortex (−1 to −4 from Bregma) was snap‐frozen in liquid nitrogen. The rest of the brain was fixed in 10% formalin for 3 days at 4°C, cryoprotected in 20% glycerol and stored at −80°C thereafter. Coronal sections (10 µm) were prepared form the caudal part of the brain.

Blood sampling was done according to previously described guidelines (60). Briefly, rats were anesthetized with 5% isoflurane and blood was collected from the tail vein prior to TBI and 2 weeks post‐TBI. Hemolyzed samples were excluded from further analysis.

### Size‐exclusion chromatography (SEC) of plasma

SEC analysis was performed as previously described (8, 21). Briefly, plasma samples (200 µL) from four rats (two TBI, one sham and one naïve rat) were passed through sepharose CL‐2B (GE Healthcare; Uppsala, Sweden) chromatography columns. A total of 25 fractions of 0.5 mL each were collected.

### Plasmids

MiRNA expressing vectors were prepared by cloning the DNA fragments encoding stem‐loop pre‐miRNA sequences including their ~100‐200 bp flanking regions into the multiple cloning site of a pCDH‐EF1a plasmid with green fluorescent protein from copepod Pontellina plumata (copGFP) as reporter (a kind gift from Dr. J. Kluiver and Dr. J. Guikema, Amsterdam UMC, Amsterdam, the Netherlands) using NheI and NotI restriction sites. The DNA fragments were amplified from human genomic DNA for hsa‐mir‐142 and from C. elegans genomic DNA for cel‐mir‐59, used as a negative control (NC).

### Cell culture

The culture medium contained Dulbecco's Modified Eagle Medium (DMEM) for HEK 293T cells, DMEM/F10 Ham (1:1) for astrocytes or Roswell Park Memorial Institute (RPMI) 1640 for THP‐1 cells (Gibco/Thermo Fisher Scientific, Waltham, MA, USA), supplemented with 2 mM L‐glutamine, 100 units/mL penicillin, 100 µg/mL streptomycin and 10% heat‐inactivated fetal calf serum (FCS) (Gibco, Life Technologies, Grand Island, NY, USA).

A HEK 293T cell line was used to produce lentiviral particles: 6.8 µg plasmid DNA, 1.7 µg pMD2.G/VSV‐G (Addgene plasmid #12259) and 3.4 µg psPAX2 (Addgene plasmid # 12260) DNA using Genius transfection reagent (Westburg, Leusden, the Netherlands) according to the manufacturer's instructions.

The human monocytic cell line THP‐1 was used to generate cell lines overexpressing hsa‐mir‐142 and cel‐miR‐39. The cells expressing copGFP were sorted on a Sony SH800S cell sorter (San Jose, CA, USA). Further, THP‐1 cells were used to produce macrophage‐conditioned medium (MCM). Briefly, THP‐1 cells (1.5 × 10^6^ per well in 6‐well plates) were differentiated into macrophage‐like cells by stimulation with 80 nM phorbol 12‐myristate 13‐acetate (PMA; Sigma‐Aldrich, St. Louis, MO, USA) for 12 h, followed by 24 h incubation with fresh medium. To induce a pro‐inflammatory state, the cells were stimulated with 10 ng/mL lipopolysaccharide (LPS from E. coli O55:B5, Sigma‐Aldrich, St. Louis, MO, USA) for 1 h, washed twice, incubated with fresh medium (2 mL per well) for 24 h and the supernatants were collected. The MCM was centrifuged at 1500 × *g* for 5 minutes and filtered through a 0.22 µm filter. Levels of TNF‐α were measured in culture supernatants (MCM) using the PeliKine Compact TNF‐α ELISA kit (Sanquin, Amsterdam, the Netherlands) according to the manufacturer's instructions.

Human primary fetal astrocyte‐enriched cell cultures were isolated and maintained as previously described (23). For treatment with MCM, astrocytes were seeded as 50 000 cells per well in 12‐well plates, allowed to attach for 24 h and stimulated MCM/fresh medium 1:1 mix. Cells were incubated for 6 h and harvested for RNA extraction. For LPS‐stimulated MCM, astrocytes were additionally treated with 100 ng/mL of Toll‐like receptor (TLR) 4 antagonist LPS‐rs (LPS from Rhodobacter sphaeroides; Invivogen, Toulouse, France).

### RNA extraction

RNA isolation from rat cortex (5–30 mg) was done using phenol/chloroform extraction following the previously described protocol (39). RNA isolation from rat plasma (100 µL), human cortex (30–50 mg) and human primary cells was done using the miRNeasy Mini kit (Qiagen Benelux, Venlo, the Netherlands) according to the manufacturer's instructions. A spike‐in exogenous control (5.6 × 10^8^ copies/mL of cel‐miR‐39‐3p; miRNeasy Serum/Plasma Spike‐In Control Cat. #219610, Qiagen Benelux, Venlo, the Netherlands) and carrier RNA from bacteriophage MS2 (1 µg/mL; Roche) were added during RNA isolation from plasma and primary human cells for subsequent normalization of RT‐qPCR data. RNA isolation from cell culture material was done using the standard phenol/chloroform isolation procedure. The concentration and purity of RNA were determined using a Nanodrop 2000 spectrophotometer (Thermo Fisher Scientific, Wilmington, DE, USA). The Qubit microRNA Assay Kit (#Q32880, Thermo Fisher Scientific) was used to measure RNA concentration in samples obtained by SEC. The protein concentration in the fractions was determined by a Pierce BCA protein assay kit (#23225, Thermo Fisher Scientific) according to the manufacturer's instructions.

### Reverse transcription (RT)

For the analysis of mRNA expression, 250 ng of total RNA was reverse‐transcribed using oligo‐dT primers. For the analysis of miRNA expression, the TaqMan miRNA RT Kit (#4366596, Applied Biosystems, Foster City, CA, USA) was used with the following primers: rno‐miR‐155‐5p (assay #002571), hsa‐miR‐155‐5p (assay #002623), hsa‐miR‐142‐3p (assay #000464), hsa‐miR‐142‐5p (assay #002248) and cel‐miR‐59‐3p (assay #001362). For normalization of data, the expression of U6 small nuclear RNA (RNU6) (assay #001973), cel‐miR‐39‐3p (assay #000200) and hsa‐miR‐23a‐3p (assay #000399) was evaluated.

### Quantitative polymerase chain reaction (qPCR)

To evaluate mRNA expression, qPCR was performed using SYBR Green I (Roche Applied Science, Indianapolis, IN, USA). The primers used are listed in Table 2. The expression was normalized using the geometric mean of *Gapdh* and *Tbp* expression in the rat brain tissue and the geometric mean of *EF1a* and *GAPDH* in the human tissue.

**Table 2:** List of oligonucleotides.

Gene symbol	Gene name	Forward	Reverse
*Rat primers*			
*Il1b*	Interleukin 1 beta	AAAAATGCCTCGTGCTGTCT	TCGTTGCTTGTCTCTCCTTG
*Tnf*	Tumor necrosis factor alpha	GCCTCTTCTCATTCCTGCTC	CTCTGCTTGGTGGTTTGCTAC
*Tgfb1*	Transforming growth factor beta 1	CCTGGAAAGGGCTCAACAC	CAGTTCTTCTCTGTGGAGCTGA
*Gfap*	Glial fibrillary acidic protein	TTTCTCCAACCTCCAGATCC	TCTTGAGGTGGCCTTCTGAC
*Pdgfrb*	Platelet derived growth factor receptor beta	TTCCTGGAGGGGGTGATAG	GGCATCACCTCTGGAAGC
*Gapdh*	Glyceraldehyde 3‐phosphate dehydrogenase	ATGACTCTACCCACGGCAAG	TACTCAGCACCAGCATCACC
*Tbp*	TATA‐Box binding protein	CAGGAGCCAAGAGTGAAGAAC	AGGAAATAACTCTGGCTCATAACTACT
*Human primers*			
*IL1B*	Interleukin 1 beta	GCATCCAGCTACGAATCTCC	GAACCAGCATCTTCCTCAGC
*TNF*	Tumor necrosis factor alpha	CCCCAGGGACCTCTCTCTAA	CAGCTTGAGGGTTTGCTACA
*PTGS2*	Prostaglandin‐endoperoxide synthase 2	GAATGGGGTGATGAGCAGTT	GCCACTCAAGTGTTGCACAT
*EF1a*	Elongation factor 1 alpha	ATCCACCTTTGGGTCGCTTT	CCGCAACTGTCTGTCTCATATCAC
*GAPDH*	Glyceraldehyde 3‐phosphate dehydrogenase	AGGCAACTAGGATGGTGTGG	TTGATTTTGGAGGGATCTCG
*Cloning primers*			
*hsa‐mir‐142*	TAAGCAgctagcAGGGAGGTAGAGGAGGCAAG	TGCTTAgcggccgcCACGTACCATCCCTTCCCAC	
*cel‐mir‐59*	TAAGCAgctagcTACACATGGCGCCAATAAAA	TGCTTAgcggccgcTTGAAAACTCTCGCTTACCG	
*In situ* *hybridization probes*			
miR‐155‐5p	5′DIG‐*AmCmC*CmCmU*AmUmC*AmCmG*AmUmU*AmGmC*AmUmUmA*A‐DIG3′		
miR‐142‐3p	5′DIG‐*TmCmC*AmUmA*AmAmG*TmAmG*GmAmA*AmCmA*CmUmAmC*A‐DIG3′		

Oligonucleotide probes for ISH had the following modifications: * = locked nucleic acid (LNA) modification; m = 2‐o‐methyl modification; DIG = digoxygenin label.

MiRNA expression was evaluated by the TaqMan microRNA assay (Applied Biosystems, Foster City, CA, USA) according to the manufacturer's instructions. The expression was normalized using the geometric mean of hsa‐miR‐23a‐3p and cel‐miR‐39‐3p in plasma, RNU6 in brain tissue and culture samples and to the cel‐miR‐39‐3p across various primary cell cultures.

RT‐qPCRs for all samples were performed in triplicates. The PCRs were run on the Roche LightCycler 480 (Roche Applied Science, Basel, Switzerland) with a 384‐multiwell format. Data analysis were performed using LinRegPCR software (46) as previously described (24).

### Droplet digital PCR (ddPCR)

miRNA expression in the combined adjacent SEC fractions (n = 12) was evaluated with ddPCR. Reaction mixtures were prepared as described in Bio‐Rad's ddPCR Applications Guide (Bio‐Rad, http://www.bio‐rad.com/) and as previously described (61). The PCR reaction was performed in 96‐well plates using the PTC‐200 Thermal Cycler (MJ Research, St. Bruno, Canada). The fluorescence was measured with a QX100 Droplet Reader (Bio‐Rad Laboratories Inc., Hercules, CA, USA) and analyzed with QuantaSoft software v1.7 (Bio‐Rad). All samples were run in duplicates.

### In situ hybridization (ISH)

ISH was performed as previously described (23). The oligonucleotide probes (RiboTask ApS, Odense, Denmark; Table 2) containing digoxygenin (DIG) labels, were hybridized (100 nM for miR‐155‐5p and 250 nM for miR‐142‐3p) for 1 h at 58 °C. Hybridization was detected with a sheep alkaline phosphatase (AP)‐labeled anti‐DIG antibody (1:1500, Roche Applied Science, Basel, Switzerland). Nitro‐blue tetrazolium chloride (NBT)/5‐bromo‐4‐chloro‐3'‐indolyphosphate p‐toluidine salt (BCIP) was used as chromogenic substrate for AP (1:50 diluted in NTM‐T buffer: 100 mM Tris, pH 9.5; 100 mM NaCl; 50 mM MgCl_2_; 0.05% Tween 20).

### ISH with immunohistochemistry

For double‐labeling after ISH, slides were incubated for 1 h at room temperature with the following primary antibodies: mouse anti‐glial fibrillary acidic protein (GFAP; 1:4000, Sigma‐Aldrich, St. Louis, MO, USA), mouse anti‐NeuN (1:2000, MAB377, Chemicon, Temecula, CA, USA), rabbit anti‐ionized calcium binding adaptor molecule 1 (Iba1; 1:2000, Wako Chemicals, Neuss, Germany), mouse anti‐human leukocyte antigen (HLA‐DR/DP/DQ; 1:100, clone CR3/43 Agilent, Santa Clara, CA, USA), rabbit anti‐transmembrane protein 119 (TMEM119; 1:500, #HPA051870, Sigma‐Aldrich, St. Louis, MO, USA), mouse anti‐CD68 (1:200, clone KP1, Dako, Glostrup, Denmark) or rabbit anti‐CD8 (1:200, #7103, Dako, Glostrup, Denmark). Secondary horseradish peroxidase (HRP)‐conjugated antibodies (Brightvision plus kit, ImmunoLogic, Duiven, the Netherlands) were used. The visualization of the antibody‐antigen binding was done using 3‐amino‐9‐ethylcarbazole (AEC; Sigma‐Aldrich, St. Louis, MO, USA).

### Statistical analysis

Statistical analyses were performed using Graphpad prism 5. The Mann‐Whitney U‐test or Kruskal‐Wallis nonparametric test with Dunn's post hoc test were used for comparisons between groups. A value of *P* < 0.05 was assumed to indicate significant difference.

## Results

### Increased expression of miR155 in the rat cortex post‐TBI

**Figure 1:**
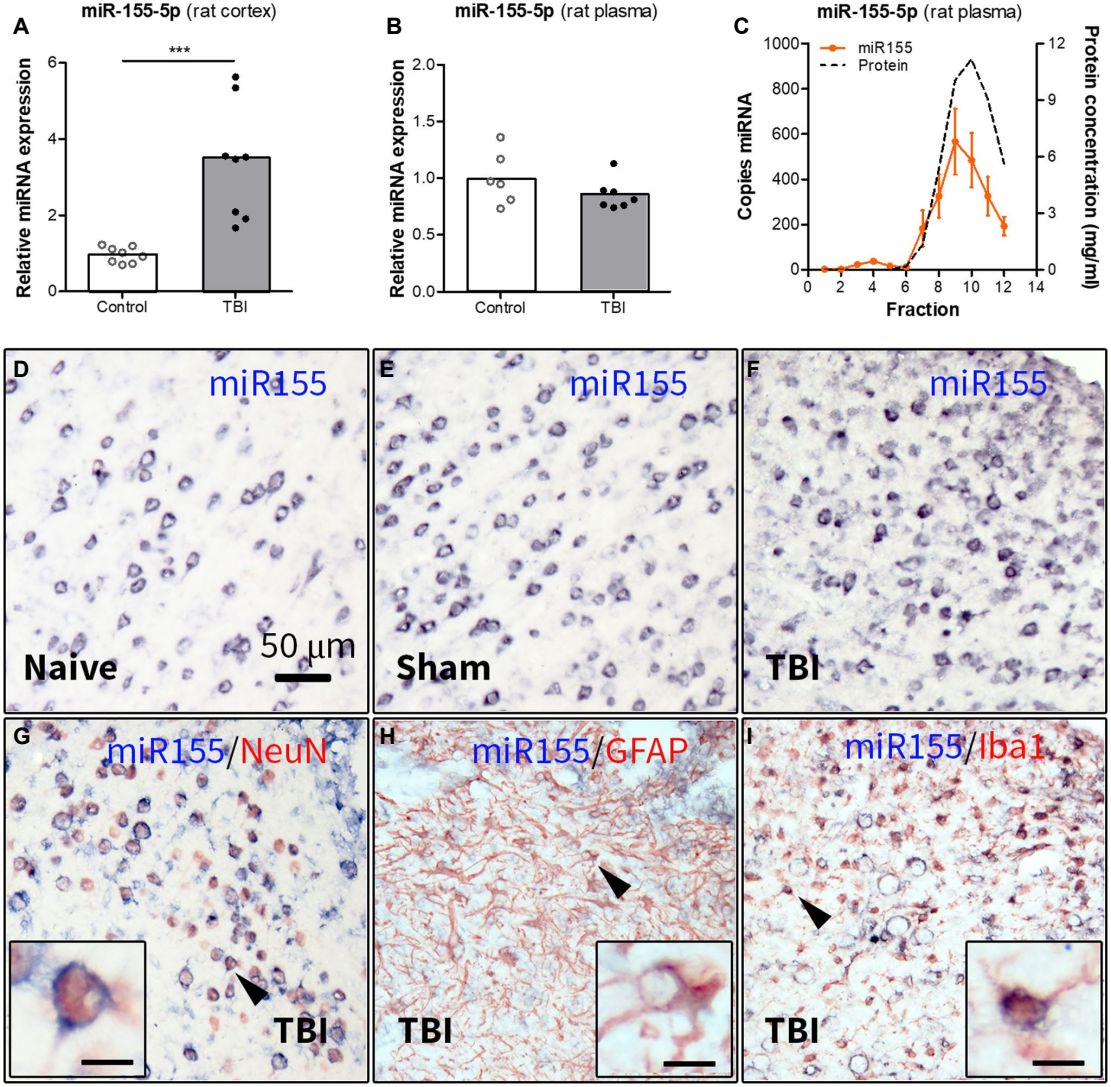
Expression of miR155 in the perilesional cortex and plasma of rats 2 weeks post‐TBI. (A) TaqMan RT‐qPCR analysis demonstrated a higher expression of miR‐155‐5p (*P* < 0.001) in the rat cortex post‐TBI as compared to (naïve + sham) controls; (B) expression of miR‐155‐5p in rat plasma post‐TBI: TaqMan RT‐qPCR analysis did not show any differences in miR‐155‐5p expression in rat plasma 2 weeks post‐TBI compared to controls; (C) ddPCR analysis of the plasma fractionated using SEC showed that miR‐155‐5p was enriched in the late‐eluting fractions, associated with proteins (n = 4 rats, SEM); (D‐I) ISH: the miR‐155‐5p hybridization signal was higher in the perilesional cortex (F) as compared to naive (D) and sham‐operated (E) rats; (G‐I) double‐labeling of the miR‐155‐5p hybridization signal with cell‐type specific markers showed co‐localization with NeuN (G), GFAP (H) and Iba1 (I) in the perilesional cortex; black arrowheads indicate the cells shown in higher magnification in corresponding insets; scale bar in D = 50 μm applies to D‐I, scale bars in insets = 10 μm; ***P < 0.001; Mann‐Whitney U‐test.

We investigated the expression of miR155 in the perilesional cortex of rats 2 weeks post‐TBI in the lateral FPI model. TaqMan RT‐qPCR analysis showed higher expression of miR‐155‐5p (Figure 1A, *P* < 0.001) in post‐TBI rats as compared to control rats.

### Circulating miR‐155‐5p is associated with proteins, rather than extracellular vesicles (EVs)

Next, we assessed the expression of miR155 in rat plasma 2 weeks post‐TBI. RT‐qPCR analysis did not show any differences in expression of miR‐155‐5p between post‐TBI and control rats (Figure 1B). The ddPCR on plasma fractions obtained by SEC showed that miR‐155‐5p was enriched in the late‐eluting fractions, associated with proteins (Figure 1C).

### MiR155 expression is observed in neurons, astrocytes and microglia

Next, we investigated the cellular expression and distribution of miR‐155‐5p using ISH. A neuronal pattern of expression and strong hybridization signal of miR‐155‐5p were observed in naive (Figure 1D), sham (Figure 1E) and post‐TBI rats (Figure 1F). Double‐labeling showed miR‐155‐5p expression in neurons (Figure 1G), astrocytes (Figure 1H) and microglia (Figure 1I) located in close proximity to the lesion edge.

### Increased miR155 expression in the white matter (WM) of the human cortex post‐TBI is associated with activated glial cells

**Figure 2:**
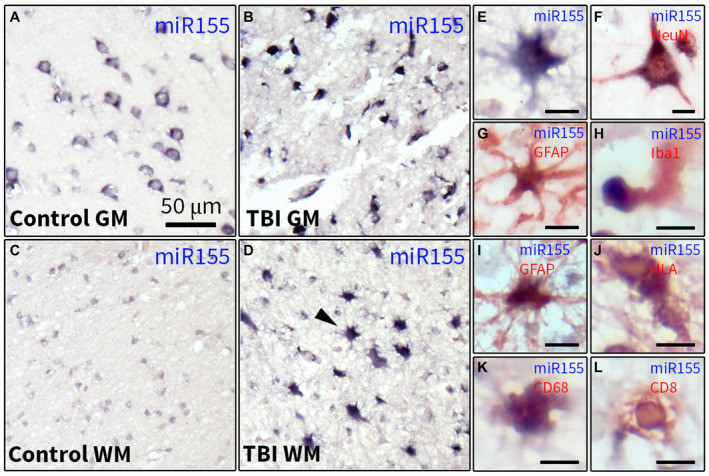
Characterization of miR155 expression in the human cortex post‐TBI. ISH of miR‐155‐5p in the GM (A, B) and WM (C, D) of the human cortex; a neuronal pattern of the miR‐155‐5p hybridization signal was observed in controls (A); the hybridization signal was stronger in individual cells in the the perilesional cortex (B); a moderate hybridization signal was detected in cells with glial morphology in the WM (C); a strong hybridization signal was observed in the WM post‐TBI (D), arrowhead indicates a cell with astrocytic morphology shown in higher magnification in E; double‐labeling showed that the miR‐155‐5p hybridization signal was co‐localized with NeuN (F), GFAP (G, I), Iba1 (H), HLA‐DP/DQ/DR (J), CD68 (K) and CD8 (L); scale bar in A = 50 μm applies to A‐D; scale bars in E‐L = 10 μm.

Next, the expression of miR‐155‐5p was analyzed in the cortex of humans post‐TBI and controls using ISH. A strong hybridization signal of miR‐155‐5p was seen in neurons in control human cortex (Figure 2A). In the perilesional cortex, neuronal loss was present; however, a strong hybridization signal was observed in all samples in the surviving neurons as well as in cells with glial morphology (Figure 2B). In the WM of controls, a moderate expression was observed (Figure 2C). In contrast, in the WM of all post‐TBI samples the ubiquitously present cells strongly expressed miR‐155‐5p (Figure 2D) and the morphology of these cells resembled astrocytes (Figure 2E). Double‐labeling showed co‐localization of miR155 with the neuronal marker NeuN (Figure 2F) in the WM and the gray matter (GM), as well as with the astrocytic marker GFAP (Figure 2G,I), the microglial markers Iba1 (Figure 2H) and HLA‐DP/DR/DQ (Figure 2J) as well as the macrophage/monocyte marker CD68 (Figure 2K) and the marker of cytotoxic T cells, CD8 (Figure 2L).

### Pro‐inflammatory conditioned medium from macrophage‐like cells upregulates miR155 in human primary astrocytes

**Figure 3:**
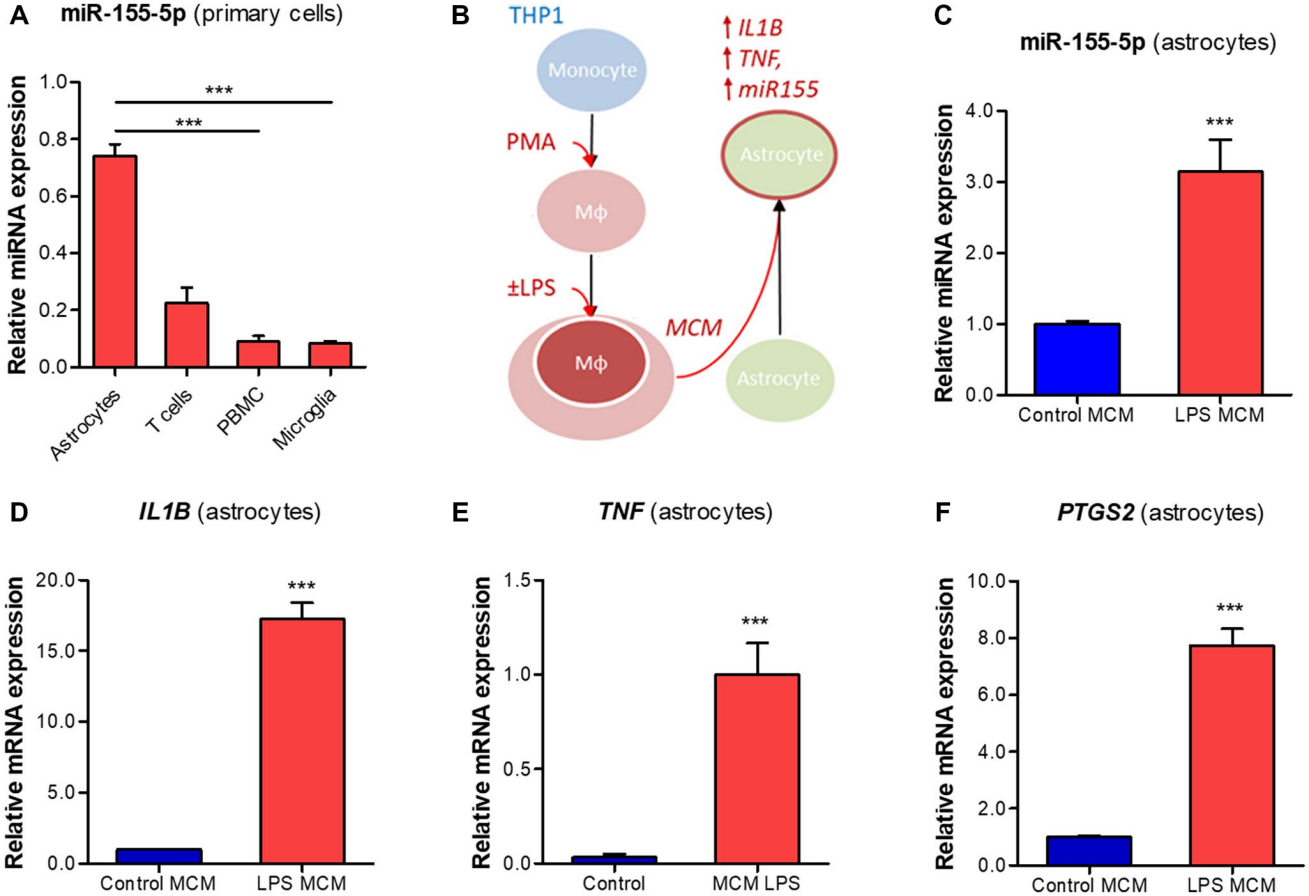
Induction of miR155 and inflammatory gene expression in human primary astrocytes by macrophage‐conditioned medium. (A) TaqMan RT‐qPCR analysis of miR‐155‐5p expression in various cell types relative to cel‐miR‐39‐3p spike‐in control: miR‐155‐5p was strongly expressed in human primary astrocytes, followed by T cells, PBMCs and microglia; (B) schematic representation of the in vitro experiment: THP‐1 cell line was converted into macrophage‐like phenotype using 80 nM PMA treatment, followed by the stimulation with 10 ng/mL LPS for 1 h and the MCM was collected 24 h later; human primary astrocytes (n = 3) were incubated with the MCM for 6 h; (C) TaqMan RT‐qPCR showed higher expression of miR‐155‐5p in astrocytes incubated with LPS‐stimulated MCM (*P* < 0.001); (D‐F) RT‐qPCR analysis of inflammatory genes in astrocytes showed an increased expression of *IL1B* (D, *P* < 0.001), *TNF* (E, *P* < 0.001) and *PTGS2* (F, *P* < 0.001); Mφ = macrophage‐like; MCM = macrophage conditioned medium; **P < 0.01, ***P < 0.001; Kruskal‐Wallis with Dunn's post‐hoc test in a; Mann‐Whitney U‐test in C‐F.

A comparison of miR155 expression between various human primary cells demonstrated the highest expression in astrocytes, followed by T cells, PBMCs and microglia (Figure 3A). This confirmed the observations *in situ*, and since miR155 was strongly expressed in activated astrocytes in the WM post‐TBI, we further investigated miR155 in human astrocytes in vitro. A higher expression of inflammatory markers, including *Il1b and Tnf was observed* in the rat perilesional cortex (Supporting Figure S1). The activated microglia and macrophages exhibiting pro‐inflammatory phenotypes may contribute to such expression profile. In order to mimic these conditions, we incubated the astrocytes with the MCM produced by PMA‐activated THP‐1 cells following LPS stimulation (Figure 3B). TaqMan RT‐qPCR showed higher expression of miR‐155‐5p in astrocytes (*P* < 0.001) following 6 h incubation with LPS‐induced MCM compared to non‐stimulated MCM (Figure 3C). This effect was the same when astrocytes were additionally treated with LPS‐rs, signifying that the increase was not mediated by any carry‐over of LPS to the MCM. We also observed higher expression of inflammatory markers: *IL1B* (Figure 3D), *TNF* (Figure 3E) and *PTGS2* (Figure 3F; all *P* < 0.001), indicating that the LPS‐stimulated MCM induced a pro‐inflammatory phenotype in human astrocytes.

### Increased expression of miR142 in the rat cortex post‐TBI

**Figure 4:**
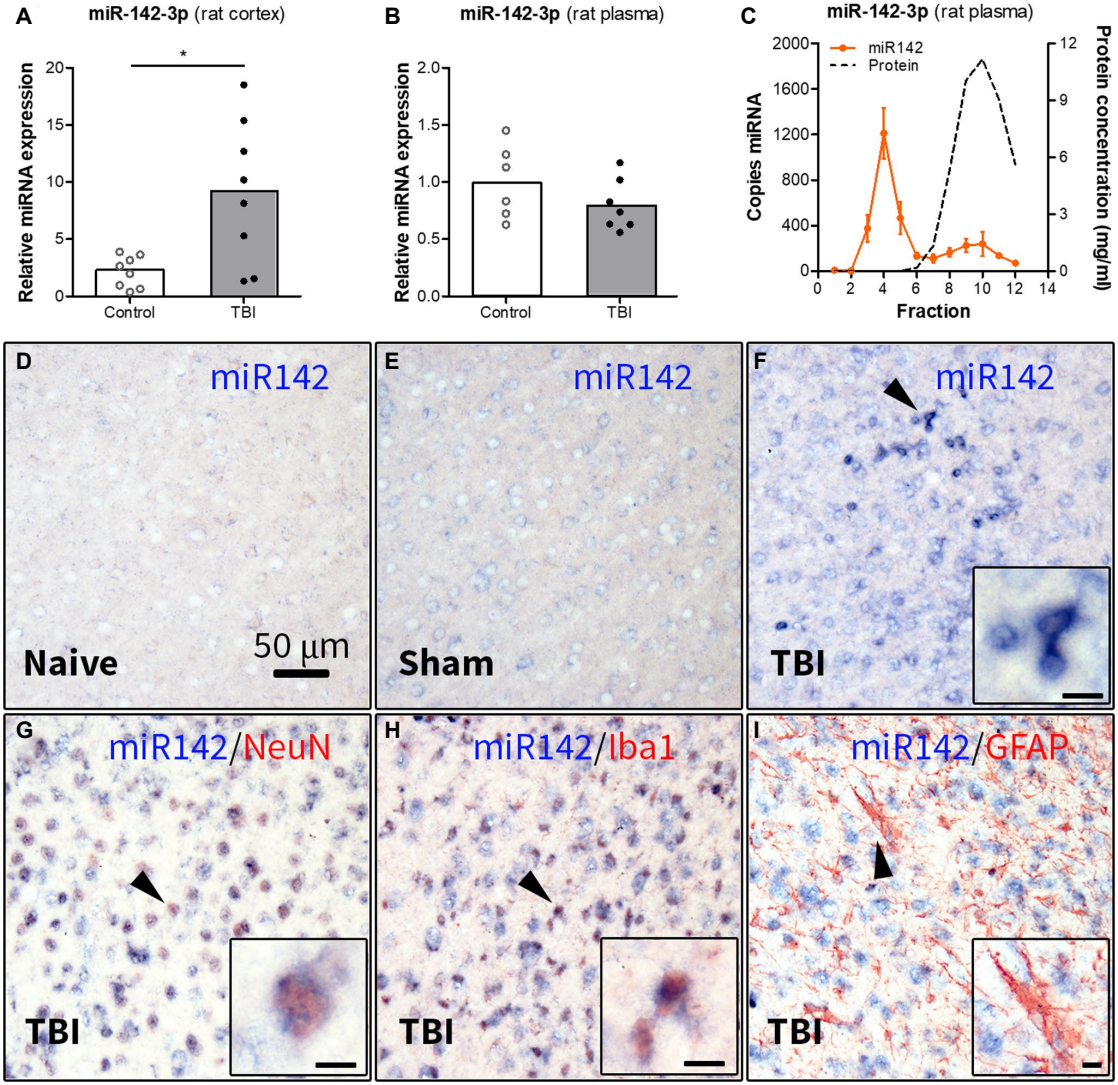
Expression of miR142 in the perilesional cortex and plasma of rats 2 weeks post‐TBI. (A) TaqMan RT‐qPCR analysis demonstrated higher expression of miR‐142‐3p (*P* < 0.05) in the rat brain cortex post‐TBI compared to the (naïve + sham) controls; (B) expression of miR‐142‐3p in rat plasma post‐TBI: TaqMan RT‐qPCR analysis did not show any difference in the miR‐142‐3p expression in rat plasma 2 weeks post‐TBI compared to controls; (C) ddPCR analysis of the plasma fractionated using SEC showed that miR‐142‐3p was enriched in the early eluting fractions, associated with EVs (n = 4 rats, SEM); (D‐I) ISH: the miR‐142‐3p hybridization signal was weak in naive (D) and sham‐operated (E) rats, but strong in the perilesional cortex (F); (G‐I) double labeling of miR‐142‐3p hybridization signal with cell‐type specific markers showed co‐localization with NeuN (G) and Iba1 (H), but not with GFAP (I); black arrowheads indicate the cells shown in higher magnification in corresponding insets; scale bar in D = 50 μm applies to D‐I, scale bars in insets = 10 μm; *P < 0.05, Mann‐Whitney U‐test.

We further investigated the expression of miR142 in the rat brain post‐TBI. RT‐qPCR analysis showed higher expression of miR‐142‐3p (*P* < 0.05, Figure 4A) and miR‐142‐5p (*P* < 0.05, Supporting Figure S2A) in the perilesional cortex of rats 2 weeks post‐TBI compared to control rats. Since both strands of the mature miR142 have been reported to be functional, we compared the relative expression of miR‐142‐3p and miR‐142‐5p. The expression of the miR‐142‐3p strand was> 200‐fold higher compared to the miR‐142‐5p in the control and post‐TBI rat brain (*P* < 0.001, Supporting Figure S2B). Therefore, we further focused on the miR‐142‐3p strand.

### Circulating miR142 is associated with EVs, rather than proteins

Next, we assessed miR142 expression in rat plasma 2 weeks post‐TBI. RT‐qPCR analysis did not show any difference in expression of miR‐142‐3p (Figure 4B) and miR‐142‐5p (Supporting Figure S2C) between post‐TBI and control rats. The expression of the miR‐142‐3p strand was ~10‐fold higher (*P* < 0.001; Supporting Figure S2D) as compared to the miR‐142‐5p strand in both control and post‐TBI rat plasma. We further assessed what type of carrier the circulating miR‐142‐3p was associated with by performing a ddPCR analysis on plasma fractions obtained by SEC. MiR‐142‐3p was enriched in the early eluting fractions, associated with EVs (Figure 4C), whereas miR‐142‐5p was predominantly enriched in the late‐eluting fractions, associated with proteins (Supporting Figure S2E).

### MiR142 expression is observed in neurons and microglia post‐TBI

Next, we investigated the cellular expression and distribution of miR‐142‐3p using ISH. The miR‐142‐3p hybridization signal was almost undetectable in the cortex of naive rats (Figure 4D), weak in sham‐operated rats (Figure 4E), but strong in the perilesional cortex (Figure 4F). Double‐labeling with cell‐type specific markers revealed that miR‐142‐3p co‐localized with the neuronal marker NeuN (Figure 4G) and the microglial marker Iba1 (Figure 4H), but did not co‐localize with a marker of astrocytes GFAP in the perilesional cortex (Figure 4I).

### Expression of miR142 in the human brain post‐TBI is associated with microglia and cells of hematopoietic origin

**Figure 5:**
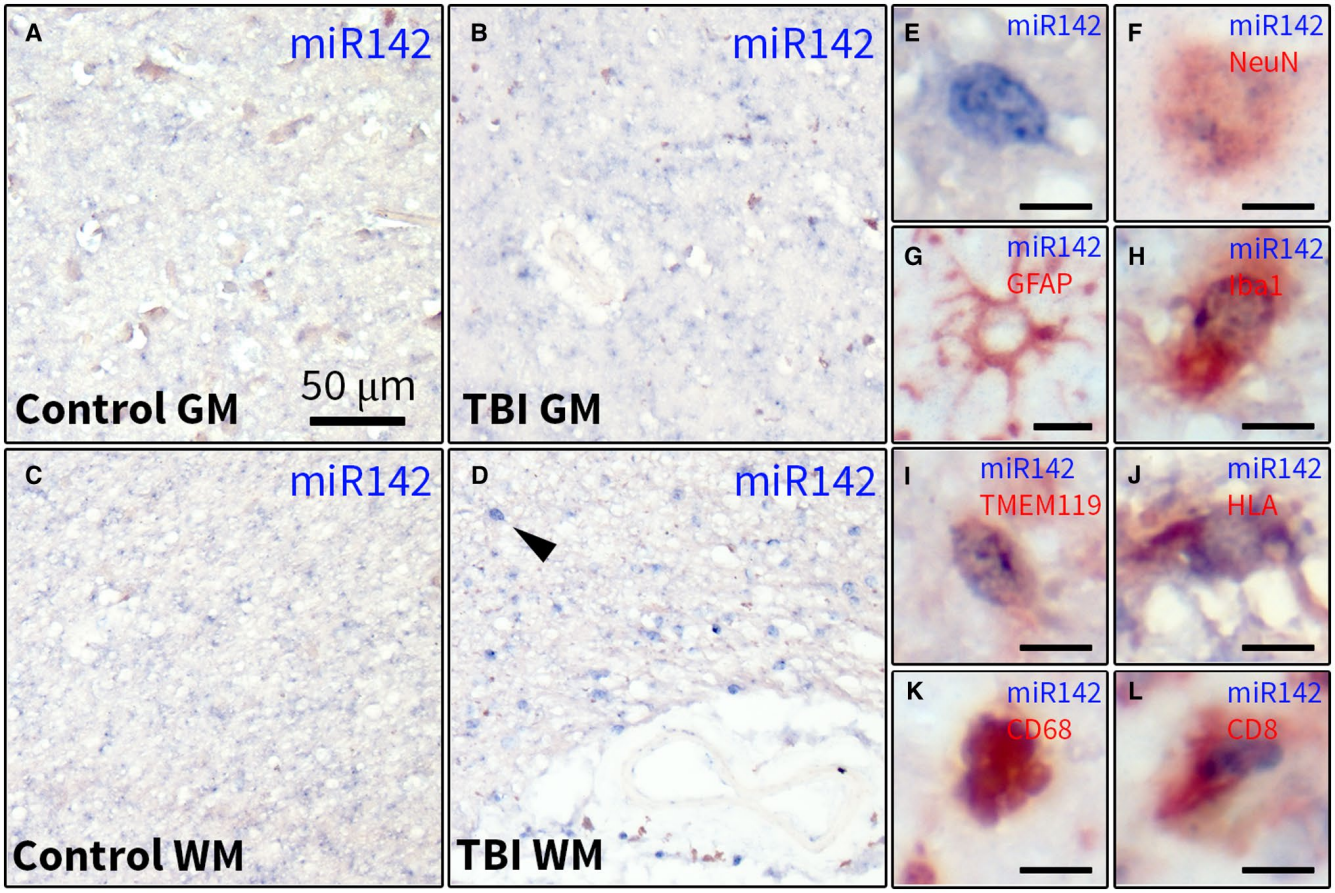
Characterization of miR142 expression in the human cortex post‐TBI. ISH for miR‐142‐3p in the GM (A, B) and WM (C, D) of the human cortex: no miR‐142‐3p hybridization signal was observed in the control human cortex (A, C); the hybridization signal was observed in cells that were sparsely present in the post‐TBI cortex (B, D), arrowhead in D indicates the cell shown in higher magnification in E; double‐labeling showed that a weak hybridization signal was co‐localized with NeuN in the GM (F); no hybridization signal was co‐localized with GFAP; the stronger hybridization signal was co‐localized with Iba1 (H), TMEM119 (I), HLA‐DP/DQ/DR (J), CD68 (K) and CD8 (L); scale bar in A = 50 μm applies to A‐D, scale bars in E‐L = 10 μm (various magnifications shown).

We further investigated miR142 expression in the human brain. Similar to the findings in the rat cortex, the expression of the miR‐142‐3p strand was higher (*P* < 0.01) than miR‐142‐5p strand in the control human cortex (Supporting Figure S2F). ISH did not reveal a hybridization signal of miR‐142‐3p in the control human cortex in the GM (Figure 5A) and WM (Figure 5C). A stronger miR‐142‐3p hybridization signal was seen in individual cells throughout the cortex of all samples post‐TBI, around the lesions in the WM, as well as in the meninges and blood vessels (Figure 5B,D,E). Double‐labeling of miR142 hybridization signal with cell‐type specific markers revealed a weak hybridization signal located in the cells expressing NeuN in the GM (Figure 5F). The hybridization signal was not co‐localized with GFAP in the GM (Figure 5G); however, a weak hybridization signal was co‐localized with GFAP in the WM astrocytes in the vicinity of lesions. The stronger hybridization signal was co‐localized with the microglial markers Iba1 (Figure 5H), TMEM119 (Figure 5I) and HLA‐DR/DP/DQ (Figure 5J). A strong hybridization signal co‐localized with the macrophage/monocyte marker CD68 in cells with macrophage morphology (Figure 5K) as well as with CD8‐positive cells inside and along the walls of blood vessels in (Figure 5L).

### miR142 overexpression in the human macrophage/microglia in vitro model increases production of TNF‐α

**Figure 6:**
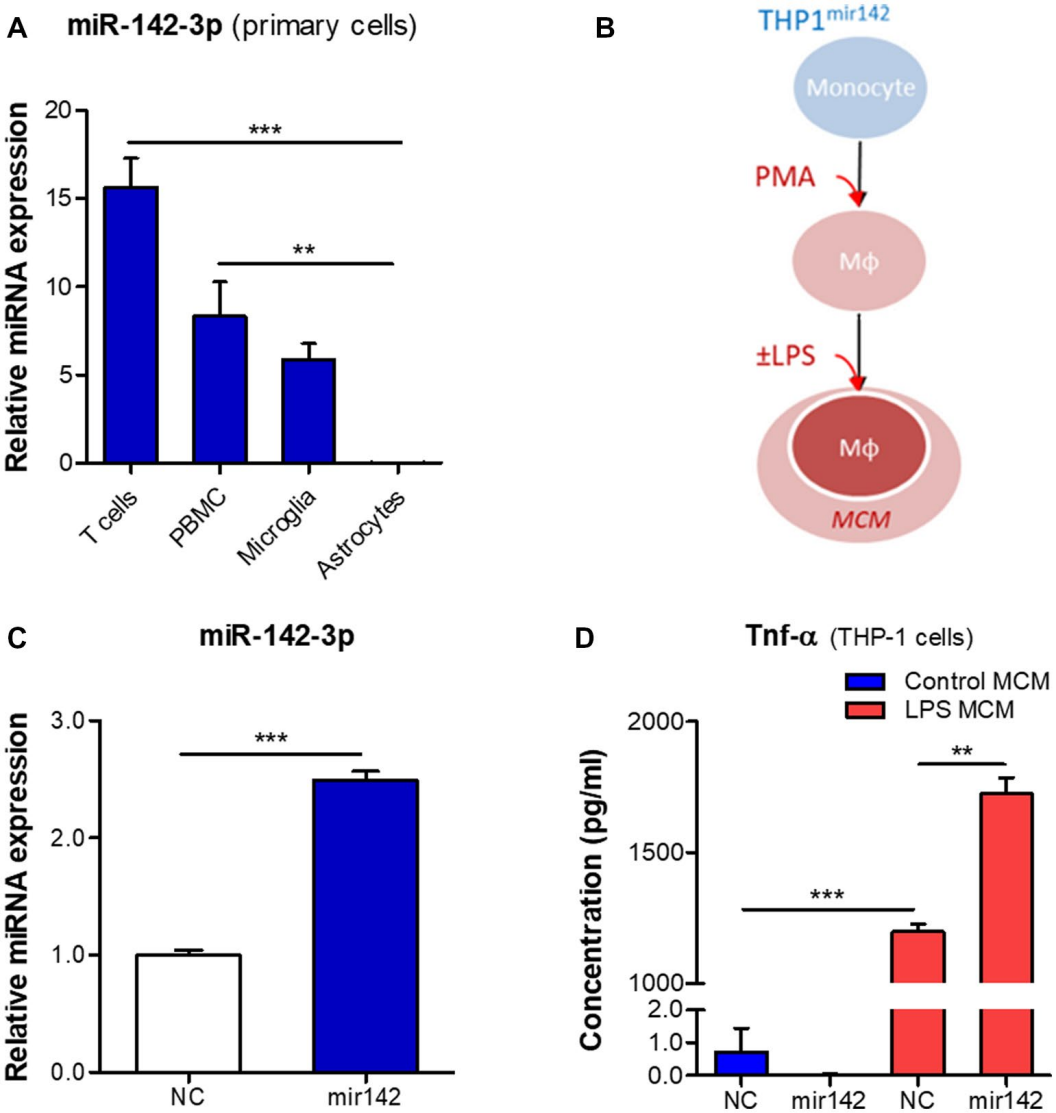
miR142 overexpression increases production of TNF‐α in THP‐1 cells. (A) TaqMan RT‐qPCR analysis of miR‐142‐3p expression in various cell types relative to cel‐miR‐39‐3p spike‐in control: miR‐142‐3p was strongly expressed in primary human T cells, PBMCs and microglia, but a relatively low expression was observed in astrocytes; (B) schematic representation of the in vitro experiment: THP‐1 cell line stably overexpressing miR142 was converted into macrophage‐like phenotype using 80 nM PMA treatment, followed by stimulation with 10 ng/mL LPS; (C) RT‐qPCR analysis showed higher basal expression of miR‐142‐3p (*P* < 0.001) in the THP1^mir142^ cells relative to the NC cells; (D) ELISA analysis showed a higher level of TNF‐α (*P* < 0.01) produced by THP1^mir142^ cells as compared to the NC cells; Mφ = macrophage‐like; MCM = macrophage conditioned medium; NC = negative control cells (THP1^celmir59^); mir142 = cells overexpressing miR142 (THP1^mir142^); **P < 0.01, ***P < 0.001; Kruskal‐Wallis with Dunn's post‐hoc test in A; Mann‐Whitney U‐test in C, D.

We further sought to identify whether increased expression of miR142 could evoke an inflammatory response in vitro. A comparison of miR142 expression between various human primary cells showed that miR142 was highly expressed in T cells, PBMCs and microglia, but lowly in astrocytes (Figure 6A, Supporting Figure S2G), corresponding with the observations *in situ*. The expression of miR‐142‐3p in PBMCs and microglia was comparable with the expression in the THP‐1 cell line (Supporting Figure S3); therefore, we used THP‐1 as a macrophage/microglia cell model to stably overexpress miR142 with the lentiviral vector (THP1^mir142^). THP‐1 cells were differentiated into macrophage‐like cells by the treatment with PMA and the pro‐inflammatory state was induced by LPS stimulation (Figure 6B). TaqMan RT‐qPCR showed higher expression of miR‐142‐3p in the THP1^mir142^ cells (*P* < 0.001) compared to the NC (THP1^celmir59^) (Figure 6C). ELISA analysis showed that the conditioned medium produced by the THP1^mir142^ cells following LPS stimulation had higher level of a major pro‐inflammatory cytokine TNF‐α (Figure 6D).

### MiR142‐overexpression in THP‐1 cells induces a pro‐inflammatory phenotype in astrocytes

**Figure 7:**
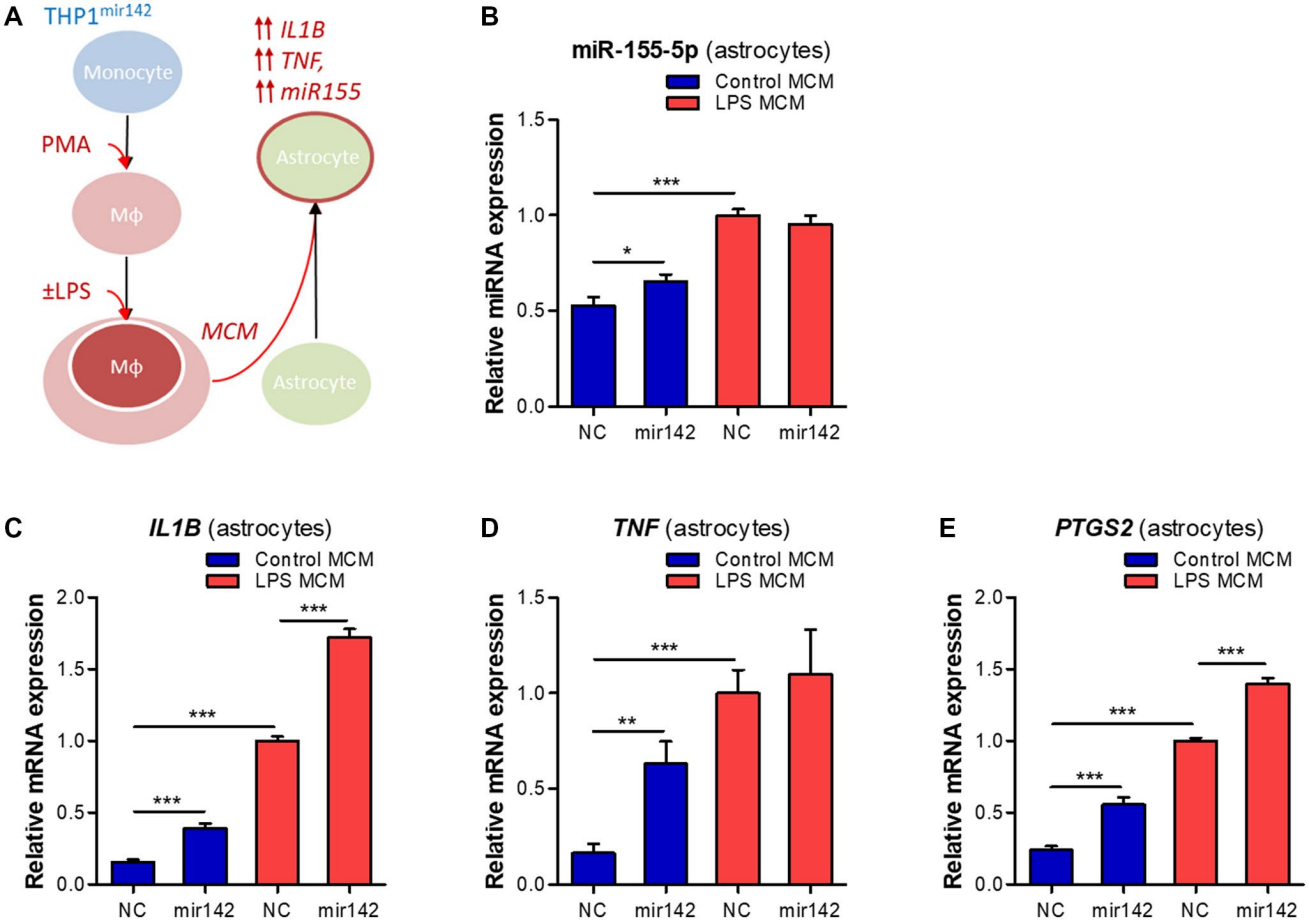
MiR142 overexpression in THP‐1 cells induces a pro‐inflammatory phenotype in human astrocytes. (A) Schematic representation of the in vitro experiment: the THP‐1 cell line was converted into macrophage‐like phenotype by a 80 nM PMA treatment, followed by stimulation with 10 ng/mL LPS for 1 h, and the MCM was collected 24 h later; human fetal primary astrocytes (n = 3) were incubated with the MCM for 6 h; (B) TaqMan RT‐qPCR showed higher expression of miR‐155‐5p in astrocytes incubated with LPS‐stimulated MCM as compared to NC (*P* < 0.001); the astrocytes incubated with THP1^mir142^ MCM showed a higher miR‐155‐5p expression as compared to the NC (*P* < 0.05); (C‐E) RT‐qPCR analysis in astrocytes showed higher expression of *IL1B* (C, *P* < 0.001), *TNF* (D, *P* < 0.01) and *PTGS2* (E, *P* < 0.001) after incubation with non‐stimulated THP1^mir142^ MCM, and further increase of *IL1B* (C, *P* < 0.001) and *PTGS2* (E, *P* < 0.001) after incubation with LPS‐stimulated THP1^mir142^ MCM; Mφ = macrophage‐like; MCM = macrophage conditioned medium; *P < 0.05, **P < 0.01, ***P < 0.001; Mann‐Whitney U‐test.

We further hypothesized that the THP‐1^mir142^ MCM could potentiate the pro‐inflammatory phenotype in human astrocytes (Figure 7A). The incubation of human astrocytes with the MCM produced by THP1^mir142^ cells led to a ~20% higher expression (*P* < 0.05) of miR‐155‐5p as compared to the cells incubated with MCM from NC cells. However, the incubation of astrocytes with the MCM produced in THP1^mir142^ cells under LPS stimulation did not change miR‐155‐5p expression compared to the NC (Figure 7B). We further assessed the inflammatory gene expression pattern in astrocytes and found higher expression of *IL1B* (Figure 7C, *P* < 0.001), *TNF* (Figure 7D, *P* < 0.01) and *PTGS2* (Figure 7E, *P* < 0.001) in cells incubated with non‐stimulated THP‐1^mir142^ MCM; as well as higher *IL1B* (Figure 7C, *P* < 0.001) and *PTGS2* (Figure 7E, *P* < 0.001), but not *TNF* (Figure 7D) in the cells incubated with LPS‐stimulated THP1^mir142^ MCM.

## Discussion

We investigated the expression of miR155 and miR142 in the perilesional cortex and plasma of rats 2 weeks post‐TBI, characterized their cell‐type specific expression in the human cortex post‐TBI and further studied these miRNAs in the human cells in vitro. Higher expression of both miRNAs in the brain post‐TBI was associated with activated glial and infiltrating immune cells. MiR155 and inflammatory genes were induced in astrocytes by a pro‐inflammatory macrophage conditioned medium (MCM). This effect was further potentiated by the MCM from the cells overexpressing miR142, suggesting that miR142 may promote neuroinflammation post‐TBI. These results are discussed in more detail in the following paragraphs.

### Increased expression of miR155 and miR142 in the cortex post‐TBI

Increased expression of miR142 and miR155 has been previously reported in the CCI post‐TBI model (16, 27, 28, 31, 44, 54, 62). We corroborated these findings for both miRNAs in the perilesional cortex of rats after lateral FPI. The two miRNAs have different expression patterns, with miR142 virtually absent in control rat brain, but present in neurons and microglia post‐TBI, and miR155 ubiquitously expressed in both control and post‐TBI brain. The two miRNAs are important regulators of the innate immune response and inflammation (14, 48), and their upregulation 2 weeks after the injury may indicate that they participate in the sustained inflammatory response.

Furthermore, the expression of these miRNAs in the human perilesional cortex resembles the patterns observed in the rat brain after TBI. Since the number of human TBI samples used for ISH is rather low we need to interpret these results with caution. Moreover, miR142 and miR155 expression was also validated by PCR using fresh brain tissue and in vitro by studying their expression in cultured cells. Altogether, this gives a complete and consistent picture. MiR155 is expressed only moderately by glial cells in the healthy cortex; however, a strong activation could be observed post‐TBI, especially in hypertrophic astrocytes in the WM. MiR142 could not be detected in the cortex of controls, recapitulating the observations from the rat brain. However, miR142‐positive cells could be observed in the cortex post‐TBI. These cells co‐expressed the markers of microglia, which is in accordance with previous observations of miR142 expression in the macaque and rat microglia (9, 33). Furthermore, we observed miR142 co‐localization with the markers of macrophages and cytotoxic T cells, which is because of the enrichment of miR142 in the cells of hematopoietic origin (10).

The increased expression of the two miRNAs has been previously shown in several brain pathologies, all associated with strong inflammation in the brain, such as multiple sclerosis (2, 20) and viral encephalitis (37) as well as temporal lobe epilepsy (3, 18, 24) and tuberous sclerosis complex (5, 47). Therefore, since miR155 is overexpressed in activated astrocytes and miR142—in myeloid and lymphoid cells, these miRNAs may be involved in the regulation of brain inflammation after TBI.

### MiR155 and miR142 may contribute to astrocyte reactivity post‐TBI

Astrocytes are important mediators of the innate immune response and inflammation in the brain (11, 29, 51). The activation of astrocytes can be induced by pro‐inflammatory signals from activated microglia (30). In a similar manner, we observed that miR155 could be induced in astrocytes by the pro‐inflammatory medium from macrophage‐like cells. This increase was further potentiated when the astrocytes were incubated with the medium from cells overexpressing miR142. These cells produce increased levels of TNF‐α, which, upon acting on astrocytes, activates the pro‐inflammatory signaling pathways associated with the nuclear factor kappa‐light‐chain‐enhancer of activated B cells (NF‐κB) and activator protein 1 (AP‐1) transcription factors to induce miR155 expression (52, 55). Indeed, the expression of pro‐inflammatory genes, such as *IL1b* and *PTGS2*, was even higher in astrocytes following the stimulation by the medium from the miR142‐overexpressing cells. This suggests that miR142‐overexpressing cells observed in the brain post‐TBI may promote a pro‐inflammatory state in surrounding astrocytes. Previous evidence corroborates the idea that miR142 promotes inflammation in the brain, since miR142 knock‐out eliminated inflammation and neurological impairment in the cerebellum of mice following experimental autoimmune encephalytis (33), and miR142 expression was associated with the cytotoxic macrophages in glioblastoma (65). The astrocytes activated by the pro‐inflammatory milieu secrete cytokines, chemokines, prostaglandins and proteases (58, 59), which further contribute to brain inflammation.

### Circulating miR142 and miR155 in the rat plasma post‐TBI

We did not find any biomarker potential for miR155 based on its expression in plasma post‐TBI, and previous studies using human blood post‐TBI support this conclusion (44, 56). Although an increase in miR142 was previously observed in serum 1 day after TBI in rats (6), our experiments did not show any difference in plasma that was obtained 2 weeks post‐TBI. Interestingly, we observed that miR‐142‐3p was enriched in the plasma fractions associated with EVs, rather than proteins. This is in accordance with previous studies (1, 21) and it has been shown that only a minor portion of circulating miRNAs are associated with EVs, whereas a majority of circulating miRNAs could be found as bound to Argonaute2 protein (1). EVs, containing miRNAs and mRNAs, have been hypothesized to be actively released by the cells and participate in intercellular communication (25, 57). Given the relative abundance of miR142 in the blood compared to the brain, the EV‐associated miR142 from blood may target brain cells, and vice versa the EVs containing miR142 in association with brain‐specific markers may be found in the blood after TBI, representing an interesting substrate for further investigation.

### Conclusions

Overexpression of miR155 and miR142 in the rat and human brain post‐TBI is associated with activated glial and immune cells, respectively. Human astrocytes acquire a pro‐inflammatory phenotype and overexpress miR155 upon stimulation with a pro‐inflammatory macrophage‐conditioned medium. MiR142 expression may increase the pro‐inflammatory action of macrophages, which may further potentiate astrocyte activation. We conclude that miR155 and miR142 promote neuroinflammation via astrocyte activation and may be involved in the secondary brain injury after TBI.

## Conflict of interest

The authors have no conflicts of interest to report. We confirm that we have read the Journal's position on issues involved in ethical publication and affirm that this report is consistent with those guidelines.

## Author contributions

AK, NP, JK, EAvV, AP and EA conceived and designed the experiments. AK, NP, SDG, MH and JK performed the experiments. EA, EAvV, DWMB and JJA processed human brain tissue. JvS, JJA, NV, IH provided technical support. AK, EA, JDM, NP, AP and EAvV contributed to the data interpretation and manuscript preparation. All authors read, revised and approved the final manuscript.

## Supporting information


**Figure S1.** Gene expression analysis of pathological markers in the rat cortex post‐TBI. Higher expression of Il1b, Tnf, Tgfb1, Gfap (all p<0.001), as well as Pdgfrb (p<0.05) was found in the perilesional cortex of rats; *p<0.05, ***p<0.001; Mann‐Whitney U‐test

**Figure S2.** Expression miR‐142‐5p strand. (a) – The TaqMan RT‐qPCR showed higher miR‐142‐5p expression (p<0.05) in the rat cortex post‐TBI compared to control (naive+sham); (b) –expression of miR‐142‐3p strand was higher than miR‐142‐5p strand (p<0.001) in the rat control cortex and cortex post‐TBI; (c) – miR‐142‐5p expression did not differ in the rat plasma post‐TBI compared to control; (d) – miR‐142‐3p expression was higher (p<0.01) in rat plasma compared to miR‐142‐5p; (e) – miR‐142‐5p was enriched in the late‐eluting plasma fractions associated with proteins; (f) – miR‐142‐3p was higher expressed compared to miR‐142‐5p in the control human cortex; (g) – miR‐142‐5p was highly expressed in primary human T cells, PBMCs and microglia, as well as THP‐1 cell line, but not in astrocytes; *p<0.05, **p<0.01, ***p<0.001; Mann‐Whitney U‐test in b, c, d, f; Kruskall‐Wallis with Dunn's post‐hoc test in g

**Figure S3.** Comparison of miR‐142‐3p expression between human cells. Expression of miR‐142‐3p in THP‐1 cells was lower compared to the expression in T cells (p<0.001), however did not differ from the expression in primary microglia and PBMCs and was higher than in astrocytes (p<0.001); ***p<0.001; Mann‐Whitney U‐test

## Data Availability

The data that support the findings of this study are available from the corresponding author upon reasonable request.
