# Supplementary figures and images for: Increased expression of miR142 and miR155 in glial and immune cells after traumatic brain injury may contribute to neuroinflammation via astrocyte activation
Source: Brain Pathol. 2020 Jun 26;30(5):897–912. doi: 10.1111/bpa.12865 (PMC7540383; doi:10.1111/bpa.12865)

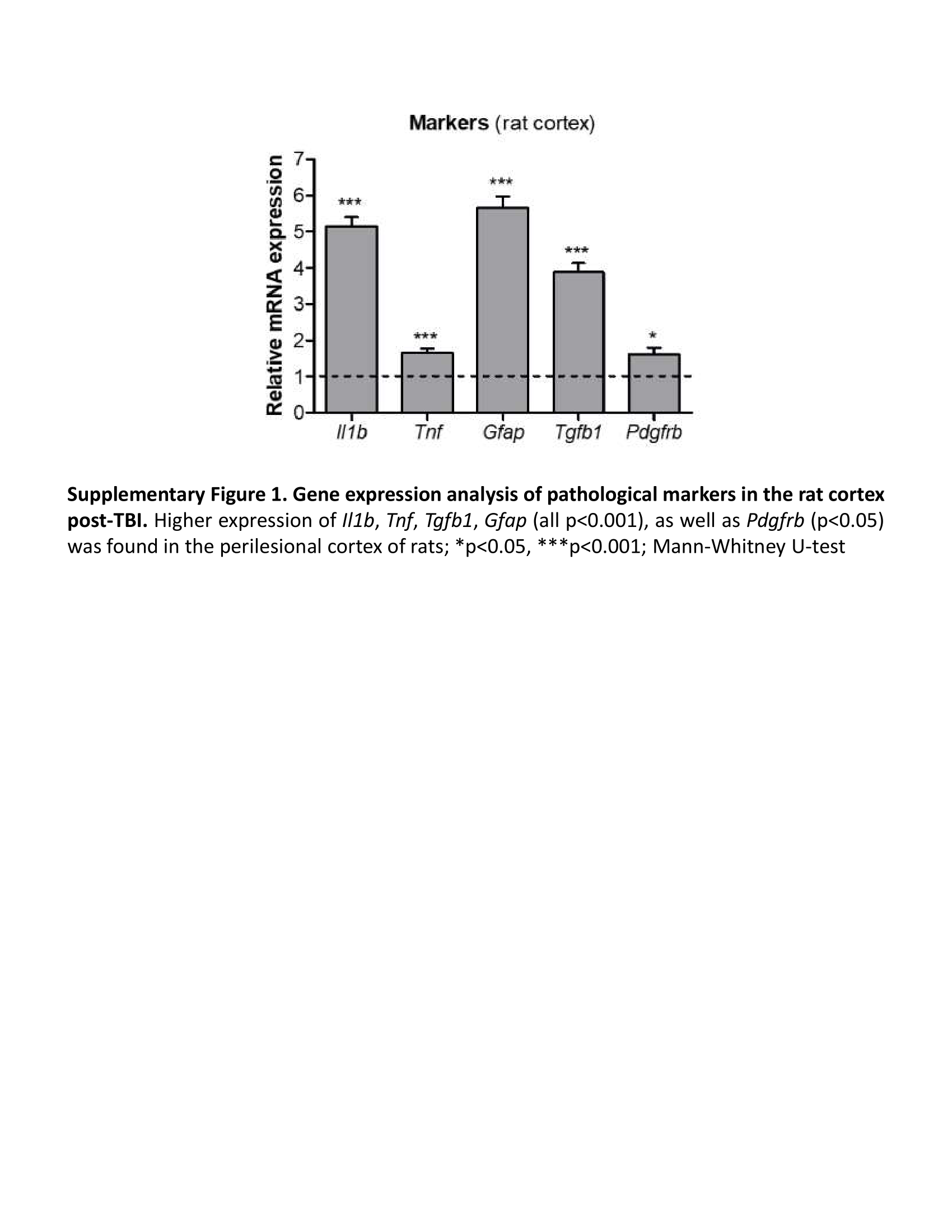

Supplement: Supplementary file 1 — Figure S1. Gene expression analysis of pathological markers in the rat cortex post‐TBI. Higher expression of Il1b, Tnf, Tgfb1, Gfap (all p<0.001), as well as Pdgfrb (p<0.05) was found in the perilesional cortex of rats; *p<0.05, ***p<0.001; Mann‐Whitney U‐test [file BPA-30-897-s003.tiff]

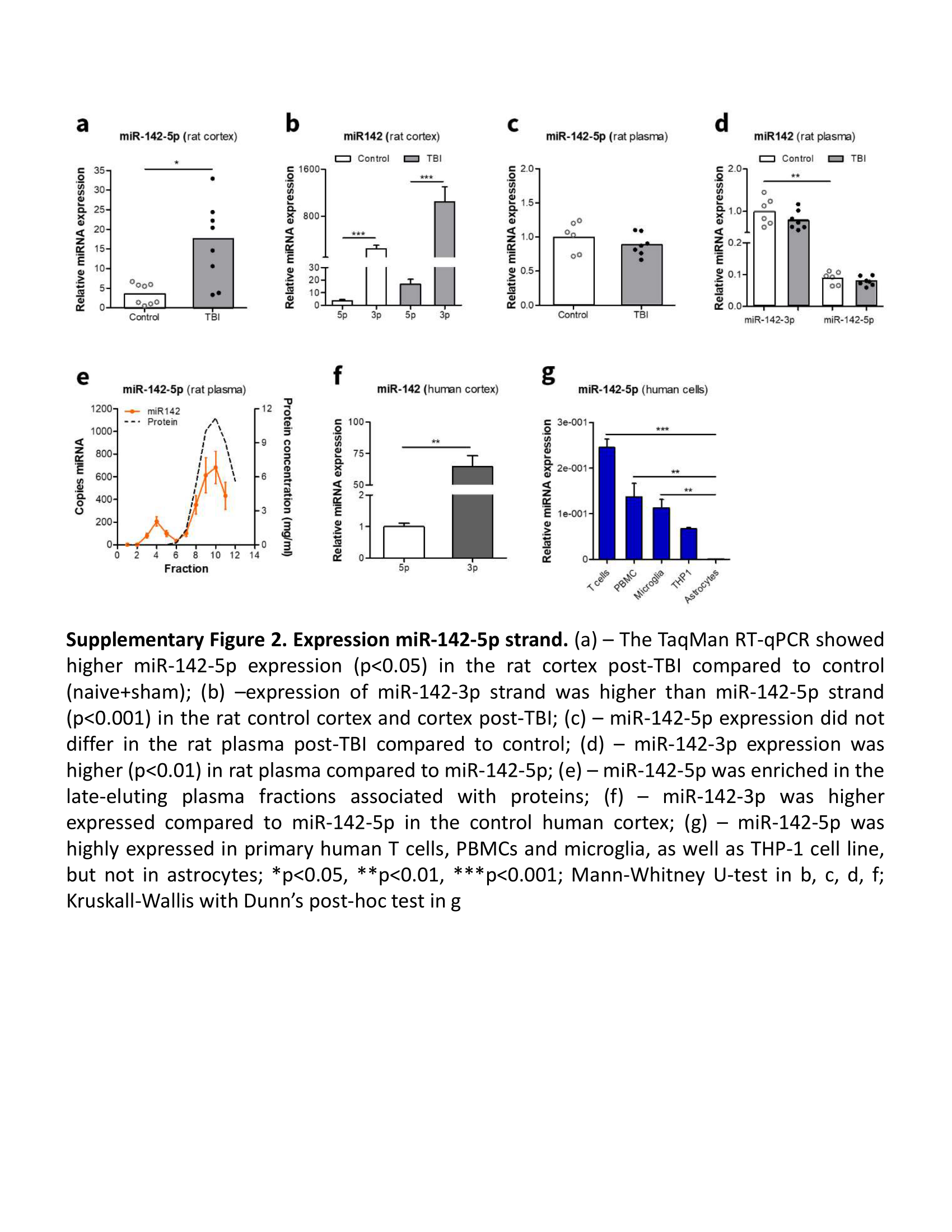

Supplement: Supplementary file 2 — Figure S2. Expression miR‐142‐5p strand. (a) – The TaqMan RT‐qPCR showed higher miR‐142‐5p expression (p<0.05) in the rat cortex post‐TBI compared to control (naive+sham); (b) –expression of miR‐142‐3p strand was higher than miR‐142‐5p strand (p<0.001) in the rat control cortex and cortex post‐TBI; (c) – miR‐142‐5p expression did not differ in the rat plasma post‐TBI compared to control; (d) – miR‐142‐3p expression was higher (p<0.01) in rat plasma compared to miR‐142‐5p; (e) – miR‐142‐5p was enriched in the late‐eluting plasma fractions associated with proteins; (f) – miR‐142‐3p was higher expressed compared to miR‐142‐5p in the control human cortex; (g) – miR‐142‐5p was highly expressed in primary human T cells, PBMCs and microglia, as well as THP‐1 cell line, but not in astrocytes; *p<0.05, **p<0.01, ***p<0.001; Mann‐Whitney U‐test in b, c, d, f; Kruskall‐Wallis with Dunn's post‐hoc test in g [file BPA-30-897-s004.tiff]

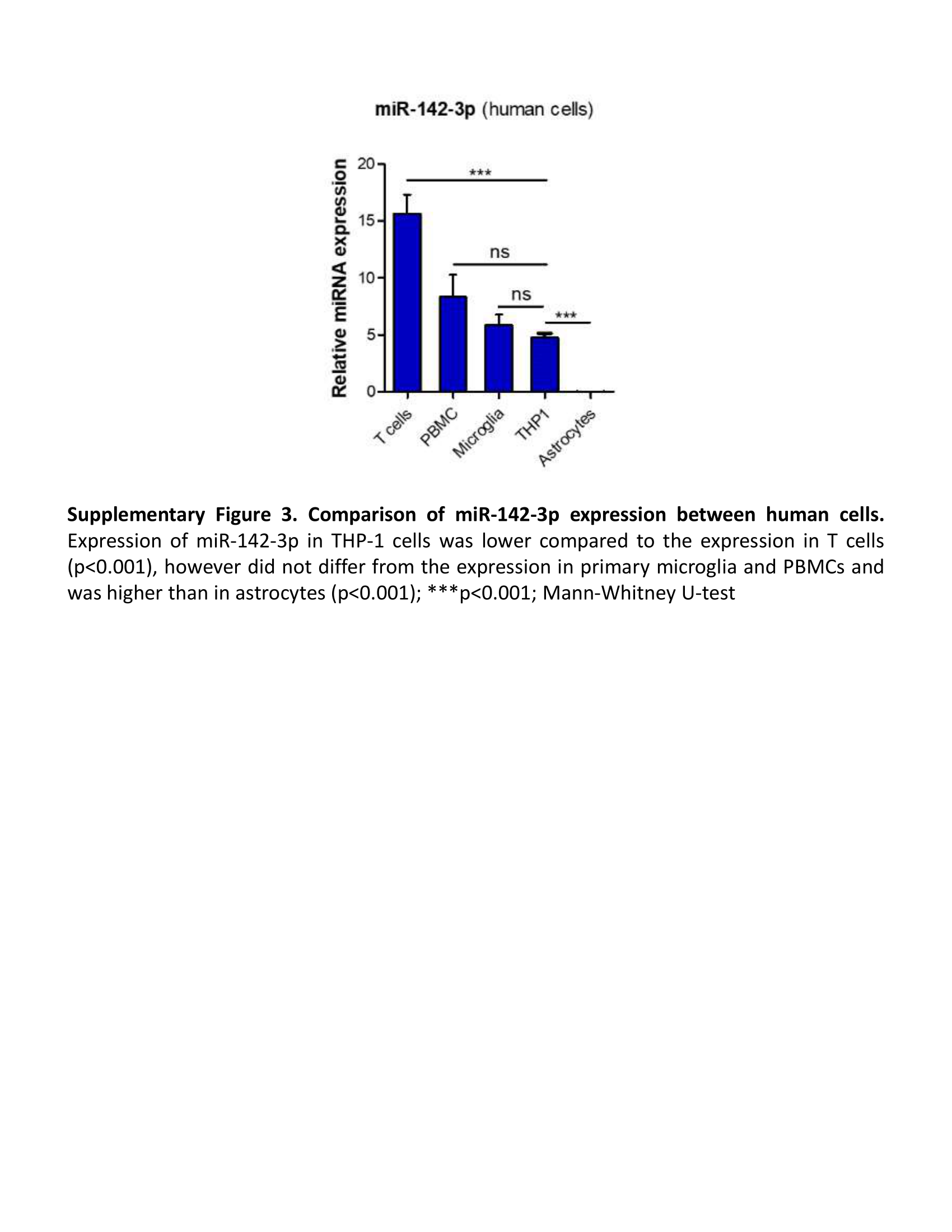

Supplement: Supplementary file 3 — Figure S3. Comparison of miR‐142‐3p expression between human cells. Expression of miR‐142‐3p in THP‐1 cells was lower compared to the expression in T cells (p<0.001), however did not differ from the expression in primary microglia and PBMCs and was higher than in astrocytes (p<0.001); ***p<0.001; Mann‐Whitney U‐test [file BPA-30-897-s002.tiff]
