# Supplementary material for: Increased expression of miR142 and miR155 in glial and immune cells after traumatic brain injury may contribute to neuroinflammation via astrocyte activation
Source: Brain Pathol. 2020 Jun 26;30(5):897–912. doi: 10.1111/bpa.12865 (PMC7540383; doi:10.1111/bpa.12865)
Supplement: Supplementary file 4 [file BPA-30-897-s001.rtf]

Supplementary Materials and methods
Increased expression of miR142 and miR155 in glial and immune cells after traumatic brain injury may contribute to neuroinflammation via astrocyte activation
A. Korotkov1, N. Puhakka2, S. Das Gupta2, N. Vuokila2, D.W.M. Broekaart1, J.J. Anink1, M. Heiskanen2, J. Karttunen2, J. van Scheppingen1,3, I. Huitinga3, J.D. Mills1*, E.A. van Vliet1,4*, A. Pitkänen2*, E. Aronica1, 5*
1Amsterdam UMC, University of Amsterdam, Department of (Neuro)Pathology, Amsterdam Neuroscience, Meibergdreef 9, Amsterdam, the Netherlands
2A. I. Virtanen Institute for Molecular Sciences, University of Eastern Finland, FI-70211 Kuopio, Finland Department of Neurology, Kuopio University Hospital, FI-70211 Kuopio, Finland.
3Department of Neuroimmunology, The Netherlands Institute for Neuroscience, Amsterdam, the Netherlands. Meibergdreef 47, 1105 BA, Amsterdam, the Netherlands
4Swammerdam Institute for Life Sciences, Center for Neuroscience, University of Amsterdam, Science Park 904, P.O. box 94246, 1090 GE, Amsterdam, the Netherlands
5Stichting Epilepsie Instellingen Nederland (SEIN), Heemstede, the Netherlands
*shared senior authors


Human brain tissue
The cases included in this study were obtained from the archives of the department of Neuropathology of the Amsterdam UMC, the Netherlands. Human post-mortem brain samples (2 males, 35 and 67 years old) who died from TBI (1-6 months post-injury) as a result of accident, without evidence of neurodegenerative changes or a clinical history of cognitive impairment, were included in this study, as well as 5 controls (3 males, 2 females, 27-71 years old) without a history of neurological diseases. Tissue was obtained and used in accordance with the Declaration of Helsinki and the Amsterdam UMC Research Code provided by the Medical Ethics Committee. All cases were reviewed by a trained neuropathologist. Brain tissue was fixed in 10% buffered formalin and embedded in paraffin.
Human primary cells
Primary fetal astrocyte-enriched cell cultures were derived from human fetal brain tissue (14–20 weeks of gestation) obtained from medically-induced abortions. All material was collected from donors from whom written informed consent for the use of the material for research purposes was obtained by the Bloemenhove clinic, the Netherlands. Tissue was obtained in accordance with the Declaration of Helsinki and the Amsterdam UMC Research Code provided by the Medical Ethics Committee. Human primary PBMCs and T cells were isolated from blood using Macs Pan T Cell Isolation Kit (Miltenyi Biotec, Bergisch Gladbach, Germany) and human primary microglia was isolated as previously described (58). More than 98% of the T cells were CD3-positive and the purity of microglia was confirmed by assessing CD45, CD11b, and CD15 expression using flow cytometry.
Animals 
Adult male Sprague-Dawley rats (n=20) (Envigo, Horst, the Netherlands) were housed in a controlled environment (temperature 22 ± 1 °C; humidity 50–60%; lights on from 07:00 to 19:00 h). At the time of experiments animals weighed (350-400 g). Water and pellet food were provided ad libitum. All animal procedures for rats were approved by the Animal Ethics Committee of the Provincial Government of Southern Finland. All animal work was carried out in accordance with the guidelines of the European Community Council Directives 2010/63/EU.
Lateral fluid-percussion injury 
The rats were subjected to lateral FPI as described previously (4, 7). The rats were randomly assigned to groups as follows: rats which received lateral FPI (n=8), sham-operated rats (n=5) and naïve rats (n=3). The rats were anesthetized by intraperitoneal injection (6 mL/kg) of a mixture of sodium pentobarbital (58 mg/kg), magnesium sulfate (127.2 mg/kg), propylene glycol (42.8%), and absolute ethanol (11.6%), and placed in a Kopf stereotactic frame (David Kopf Instruments, Tujunga, CA, USA). A midline scalp incision was made and the underlying periosteum dissected. A 5-mm circular craniectomy was performed with a trephine over the left parietal lobe, midway between lambda and bregma, with the lateral edge of the craniectomy adjacent to the lateral ridge. A modified Luer–Lock cap was cemented into the craniectomy (Selectaplus CN, Dentsply DeTRey GmbH, Dreieich, Germany) and filled with saline. At 90 min after administration of the anesthesia, animals were connected to the fluid-percussion device (AmScien Instruments, Richmond, VA, USA) through the male Luer-Lock fitting and brain injury was induced with the mean pressure (3.18 ± 0.08 atm). Duration of apnea and occurrence of acute post-impact seizures were monitored. Sham-operated control animals received anesthesia and underwent all surgical procedures without lateral FPI. The animals were sacrificed 2 weeks post-TBI. Acute mortality within 48 h post-TBI was 15% (3/20). One rat was excluded from the analysis due to the broken dura mater after TBI. 
Sample collection 
Brain samples
Briefly, rats were anesthetized with 5% isoflurane and decapitated with a guillotine. The brain was extracted and the sections at -1 – -4 Bregma were dissected into cortex, hippocampus, thalamus, hypothalamus and white matter. Both ipsilateral and contralateral parts were snap-frozen in liquid nitrogen. The rest of the brain was fixed in 10% formalin. Rostral and caudal parts of the brain were immersed in 10% formalin buffered on 0.1 M sodium phosphate buffer (PB) for 3 days on a shaking platform at 4°C, followed by a 6 days incubation in 20% glycerol buffered on potassium-phosphate buffer for cryoprotection, and subsequently frozen on dry ice-cooled metal plate and stored at -80°C thereafter. For histological analysis the caudal part of the brain was cut on a cryotome into 10 µm coronal sections, which were immediately immersed in 10% formalin and left for at least 2 days. After the incubation the sections were mounted onto Superfrost Plus slides (Thermo Scientific, Gerhard Menzel GmbH, Braunschweig, Germany) and stored at room temperature until use.  
Blood samples 
The sampling and processing of blood plasma was done according to guidelines previously described by van Vliet et al. (59). For blood collection rats were anesthetized with 5% isoflurane and blood was collected from the tail vein using a 25G butterfly needle (Surflo Winged infusion set, Terumo Europe N.V., Leuven, Belgium). The samples were collected in K2-EDTA (di-potassium ethylenediaminetetraacetic acid) tubes (Vacutainer, BD Biosciences, Franklin Lakes, NJ, USA) at 2 weeks after TBI (500 µl). The blood was centrifuged at 1,300xg within 1 h and plasma was aliquoted in 50 µL volumes and frozen at -80°C until use. To assess the level of hemolysis in the samples hemoglobin (Hb) absorbance (average value 0.21±0.08) was measured at 414 nm wavelength using Nanodrop 1000 spectrophotometer (ThermoFisher Scientific, Wilmington, DE, USA). Samples with an absorbance higher than 0.3 were considered as hemolyzed and were excluded from the further analysis. 
Plasmids
miRNA expressing vectors were prepared by cloning the DNA fragments encoding stem-loop pre-miRNA sequences including their ~100-200bp flanking regions into the multiple cloning site (MSC) of a pCDH-EF1a-MSC-copGFP (a kind gift from Dr. J. Kluiver and Dr. J. Guikema) vector using NheI and NotI restriction sites. The DNA fragments were amplified using a high-fidelity DNA polymerase Immolase (Bioline, UK, London) from human genomic DNA using the following primers for hsa-mir-142: (forward, 5'-TAAGCAgctagcAGGGAGGTAGAGGAGGCAAG-3'; reverse, 5'-TGCTTAgcggccgcCACGTACCATCCCTTCCCAC-3'). For the negative control the DNA fragment encoding cel-mir-59 was amplified from the C. elegans genomic DNA using the following primers: forward, 5'-TAAGCAgctagcTACACATGGCGCCAATAAAA-3'; reverse, 5'-TGCTTAgcggccgcTTGAAAACTCTCGCTTACCG-3'). DNA extraction from agarose gel was done with NucleoSpin Extract II Kit from Machinery-Nagel (Düren, Germany). The transformation of Stbl3 bacteria was done according to a standard procedure. The bacterial colonies were grown on LB agar plates in the presence of ampicillin (100 µg/mL). Individual colonies were selected and the presence of DNA of interest was confirmed by electrophoresis on agarose gel. Single positive colonies were inoculated in liquid culture and the plasmid DNA was isolated using NucleoBond PC100 Midi Kit from Machinery-Nagel (Düren, Germany). The concentration and quality of DNA was confirmed on Nanodrop 2000 (ThermoFisher Scientific, Wilmington, DE, USA). Sanger sequencing was used to confirm that the DNA fragments were cloned in the vector. 
Cell culture
A HEK 293T cell line was cultured in DMEM (Gibco/ThermoFisher Scientific, Waltham, MA, USA), supplemented with 2mM L-glutamine, 100 units/mL penicillin, 100 µg/mL streptomycin and 10% heat-inactivated fetal calf serum (FCS) (Gibco, Life Technologies, Grand Island, NY, USA). Lentiviral particles were produced by transfecting 6.8 µg pCDH-EF1a-mir-copGFP, 1.7 µg pMD2.G/VSVG (Addgene plasmid #12259) and 3.4 µg psPAX2 (Addgene #plasmid # 12260) DNA using Genius transfection reagent (Westburg, Leusden, the Netherlands) according to manufacturer's instructions and the cells were incubated for 24h, followed by another 24h incubation with fresh medium. The medium containing virus was harvested and filtered through a 0.22 µm filter. 
Tissue samples for primary fetal astrocyte-enriched cell cultures were collected in astrocyte medium: DMEM/HAM F10 (1:1) (Gibco/ThermoFisher Scientific, Waltham, MA, USA), supplemented with 2mM L-glutamine, 100 units/mL penicillin, 100 µg/mL streptomycin and 10% heat-inactivated fetal calf serum (FCS). Cell isolation was performed as follows: meninges and blood vessels were removed, tissue was minced and dissociated by incubation with 2.5 mg/mL trypsin at 37°C for 20 min, followed by inactivation of trypsin with astrocyte medium. The tissue was passed through a 70 µm mesh filter and the cell suspension was transferred into a flask with fresh astrocyte medium and maintained in a 5% CO2 incubator at 37°C. After 48 h incubation the medium was replaced with fresh medium and was subsequently refreshed twice a week. Cultures reached confluence after 2–3 weeks. Astrocytes were used at passages 2-5. More than 98% of the cells in primary culture, as well as in the successive passages were strongly immunoreactive for the astrocytic marker glial fibrillary acid protein (GFAP) and S100â as previously reported (60).The human monocytic cell line THP-1 was cultured in RPMI 1640 (Gibco/ThermoFisher Scientific, Waltham, MA, USA), supplemented with 2mM L-glutamine, 10% FCS, 100 units/mL penicillin, 100 µg/mL streptomycin. In order to generate monocytic cell lines overexpressing hsa-miR-142 and cel-miR-39, 0.3*106 THP-1 cells were taken up in the THP-1 medium mixed 1:1 with the medium from HEK 293T cells containing lentiviral particles with 8 µg/mL hexadimethrine bromide (Sigma-Aldrich, St. Louis, MO, USA), and the cells in 24-wells were spinofected for 60 min at 34 ˚C. The cells were incubated for 24 h, then the infected medium was removed, the cells were maintained in the THP-1 culture medium for 1 week. The cells expressing copGFP were sorted on a Sony SH800S cell sorter (San Jose, CA, USA) to create stably overexpressing cell lines.    
Macrophage conditioned medium (MCM) and ELISA assay   
MCM was produced as follows: THP-1 cells (1.5*106 per well in 6-well plates) were differentiated into macrophage-like cells by stimulation with 80 nM phorbol 12-myristate 13-acetate (PMA; Sigma-Aldrich, St. Louis, MO, USA) for 12 h, followed by another 24 h incubation with fresh THP-1 culture medium. To induce a pro-inflammatory state cell medium was replaced with the medium containing 10ng/mL lipopolysaccharide (LPS from E. coli O55:B5, Sigma-Aldrich, St. Louis, MO, USA) or fresh medium (control) for 1 h, followed by two washes with fresh medium and incubation with fresh medium (2 mL per well) for 24 h, after which supernatants were collected from individual wells and pooled together. The MCM was centrifuged at 1,500xg for 5 minutes and filtered through a 0.22 µm  filter. MCM was aliquoted and frozen at -20˚C until further use. Levels of TNF-á  were measured in culture supernatants (MCM) using the PeliKine Compact TNF-á ELISA kit (Sanquin, Amsterdam, the Netherlands) according to the manufacturer's instructions.
Treatment of astrocytes with MCM
Human primary fetal astrocytes were seeded as 50,000 cells per well in 12-well plates and allowed to attach for 24 h. The astrocyte culture medium was replaced with the fresh astrocyte medium mixed with MCM 1:1. Cells were incubated for 6 h, followed by 2 washes with warm sterile PBS and the cells were collected in 700 µL of Qiazol Lysis Reagent (Qiagen Benelux, Venlo, the Netherlands) and frozen at -20˚C until further use. In some experiments astrocytes stimulated with LPS-activated MCM were treated with LPS-rs (a TLR4 antagonist from the photosynthetic bacterium Rhodobacter sphaeroides and a competitive inhibitor of LPS activity; Invivogen, Toulouse, France; 100 ng/mL) to demonstrate that the effect of MCM were not due to a potential carry-over of LPS into MCM.
RNA extraction
RNA extraction was done from the autoptic human brain cortex, rat perilesional cortex, rat plasma and cell culture samples.
RNA was isolated from the rat brain tissue using the previously described protocol (61). Briefly, small pieces of cortex (5-30 mg) were cut and lysed in Ambion lysis buffer (Ambion, Austin, TX, USA). The tissue was mechanically homogenized in a tube with metal beads under RNase-free conditions in a cold room (-20 ˚C). The elements of Qiagen All Prep kit (Qiagen, Hilden, Germany) were used further: samples were homogenized using QIAshredder columns and DNA was removed by the AllPrep DNA spin column. The RNA was further isolated by phenol/chloroform extraction using Ambion kit (Ambion, Austin, TX, USA). 
RNA was isolated from rat plasma, human brain cortex and human primary cells using the miRNeasy Mini kit (Qiagen Benelux, Venlo, the Netherlands) according to manufacturer's instructions. For rat plasma 100 µL volume was used, diluted with by RNase-free water to obtain 200 µL. A spike-in exogenous control (5.6*108 copies/mL of cel-miR-39-3p; miRNeasy Serum/Plasma Spike-In Control Cat. #219610, Qiagen  Benelux, Venlo, the Netherlands) and carrier RNA from bacteriophage MS2 (1µg/mL; Roche) were added for subsequent normalization of RT-qPCR data. The described spike-in control was also added to a collection of human primary cell RNA samples to further normalize for miRNA expression levels between various cell types.
RNA was isolated from cell culture material using the standard phenol/chloroform isolation procedure. Briefly, 700 µL of samples lyzed in Qiazol was mixed with 140 µL chloroform, properly mixed and centrifuged at 12,000xg for 15 minutes at 4 ˚C. The aqueous phase was collected, mixed 1:1 with ice-cold isopropanol and 1 µL of glycogen blue (GlycoBlue, Thermofisher Scientific, Waltham, MA, USA) was added. The mixture was left at -20˚C overnight, followed by centrifugation at 20,000xg, 4 ˚C for 45 minutes. The RNA pellets were washed twice with ice-cold 80% ethanol and diluted in 25 µL RNase-free water.  
For size-exclusion chromatography RNA was isolated using the Qiagen miRNeasy Mini Kit (Qiagen Benelux, Venlo, the Netherlands). The adjacent fractions were combined to increase the RNA yield.
The concentration and purity of RNA were determined using a Nanodrop 2000 spectrophotometer (ThermoFisher Scientific, Wilmington, DE, USA). Qubit microRNA Assay Kit (#Q32880, Thermo Fisher Scientific) was used to measure RNA concentration in samples obtained by size-exclusion chromatography. The protein concentration in the fractions was determined by a Pierce BCA protein assay kit (#23225, Thermo Fisher Scientific) according to manufacturer instructions.
Reverse transcription (RT)
For the analysis of mRNA expression, 250 ng total RNA was reverse-transcribed using oligo-dT primers in 25 ìL of mix. The cDNA was further diluted with RNase-free water 3 times and stored at −20°C until use. For the analysis of miRNA expression in plasma, total extracted RNA was reverse-transcribed to complementary DNA (cDNA) with the TaqMan miRNA Reverse Transcription Kit (#4366596, Applied Biosystems, Foster City, CA, USA) according to the manufacturer's instructions. For miRNAs 50 ng of total RNA were reverse-transcribed using primers for rno-miR-155-5p (assay #002571), hsa-miR-155-5p (assay #002623), hsa-miR-142-3p (assay #000464), hsa-miR-142-5p (assay #002248) and cel-miR-59-3p (assay #001362). For normalization of data, the expression of U6 small nuclear RNA (RNU6) (assay #001973), cel-miR-39-3p (assay #000200) and hsa-miR-23a-3p (assay #000399) was evaluated. Reverse transcription was then performed using a T100 Thermal Cycler (Bio-Rad Laboratories Inc, Hercules, CA, USA) as follows: 16 °C for 30 min, 42 °C for 30 min, 85 °C for 5 min, and 4 °C thereafter. Samples were stored at -80 °C until further use. 
Quantitative polymerase chain reaction (qPCR) 
To evaluate mRNA expression, each qPCR reaction contained 1 µl cDNA, 2.5 µl of FastStart Reaction Mix SYBR Green I (Roche Applied Science, Indianapolis, IN, USA), 0.4 µM of both reverse and forward primers. The final volume was adjusted to 5 µl with RNase-free water. Every PCR reaction was performed in triplicates. A negative control with water instead of cDNA was included in each experiment. The cycling conditions were carried out as follows: initial denaturation at 95°C for 5 min, followed by 45 cycles of denaturation at 95°C for 15 s, annealing at 65 °C for 5 s and extension at 72 °C for 10 s. The fluorescent product was measured by a single acquisition mode at 72 °C after each cycle. The primers used for the qPCR are listed in Table 1. The geometric mean of glyceraldehyde 3-phosphate dehydrogenase (Gapdh) and TATA-box-binding protein (Tbp) expression levels was used for the normalization of RT-qPCR of mRNA expression in the rat brain tissue and the geometric mean of eukaryotic translation elongation factor 1 alpha 1 (EF1a) and GAPDH was used for human tissue. 
To evaluate mature miRNA expression Taqman microRNA assay (Applied Biosystems, Foster City, CA, USA) was used according to manufacturer's instructions. Expression of plasma miRNAs was normalized to the geometric mean of hsa-miR-23a-3p as endogenous reference and cel-miR-39-3p as exogenous reference. Expression of miRNAs in brain tissue and culture samples was normalized to RNU6 and expression of miRNAs across various primary cell cultures was normalized to cel-miR-39-3p spike-in control.
RT-qPCRs for all samples were performed in triplicates. The PCRs were run on the Roche LightCycler 480 (Roche Applied Science, Basel, Switzerland) with a 384-multiwell format. Data analysis was performed using LinRegPCR software (62) as previously described (63). 
Size-exclusion chromatography (SEC) of plasma 
SEC analysis was performed as previously described (55, 64). Briefly, the chromatography columns were prepared by loading Sepharose CL-2B (GE Healthcare; Uppsala, Sweden) into a sterile 10 mL syringe to form a column. The column was washed with at least 10 mL of filtered (0.22 µm) 0.32% trisodium citrate on PBS buffer. Pooled plasma samples (200 µL) from each of the 4 rats (2 TBI, 1 sham and 1 naïve rat) were centrifuged at 10,000xg for 5 min, the supernatant was diluted on PBS 1:1 and loaded onto the column. The elution was performed until the plasma level (visible as orange-colored band in the column) reached the end of the column, at room temperature for approximately 1 h. The column was maintained wet by adding filtered PBS 0.32% trisodium citrate buffer dropwise. A total of 25 fractions of 0.5 mL each were collected. The isolated fractions were stored at 4 °C, followed by RNA isolation or at -20 °C until use. 
Droplet digital PCR (ddPCR)
The amount of mature miRNAs in the RNA samples obtained from the combined adjacent SEC fractions (n=12) from rat plasma was evaluated with ddPCR. Reaction mixtures were prepared as described in Bio-Rad's Droplet Digital PCR Applications Guide (Bio-Rad, http://www.bio-rad.com/). Briefly, for each 20 ìL reaction, 1.33 ìL of cDNA was mixed with 1 ìL of Taqman Small RNA Assay (20×), 10 ìL of Bio-Rad's ddPCR supermix for probes, and 7.67 ìL nuclease-free water (#AM9939, Ambion, Austin, TX, USA). Samples were loaded into the middle row of DG8 Cartridges (#1864008, Bio-Rad Laboratories Inc., Hercules, CA, USA). Then, 70 ìL of Droplet Generation Oil for Probes (#1863005, Bio-Rad) was added to the bottom wells of the cartridge. The cartridge was covered with a DG8™ Gasket (#1863009 Bio-Rad) and placed into the QX200 droplet generator (Bio-Rad). After droplet generation was completed, droplets (40 ìL) were gently pipetted into the wells of a 96-well PCR plate (#951020303, Eppendorf, Hamburg, Germany), and the plate was sealed with pierceable sealing foil (#1814040 Bio-Rad) using a PX1 PCR Plate Sealer (Bio-Rad). PCR was run using the PTC-200 Thermal Cycler (ramp rate 2 °C/s; MJ Research, St. Bruno, Canada) under the following conditions: 95 °C for 10 min, then 40 cycles (15 s each) at 95 °C followed by 1 cycle at 60 °C (60 s), and finally, 98 °C for 10 min. After the PCR, the fluorescence of each droplet was measured with a QX100 Droplet Reader (Bio-Rad). The results were analyzed with QuantaSoft software v1.7 (Bio-Rad) to determine the copy number of mature miRNAs in each sample. All samples were run in duplicates. The mean amount of positive droplets across the duplicates was plotted per fraction.
In situ hybridization (ISH)
Paraffin-embedded brain tissue was deparaffinised in xylene and rinsed in ethanol (2X 100%, 1X 70%) and sterile water. Antigen retrieval was performed using a pressure cooker in sodium citrate buffer, pH 6.0, at 121°C for 10 minutes. The oligonucleotide probes that hybridize to miR-142-3p and miR-155-5p (Table 2) contained locked nucleic acid (LNA) modifications, 2-o-methyl modifications and digoxygenin (DIG) labels (RiboTask ApS, Odense, Denmark). Sections were incubated with probes (100 nM for miR-155-5p and 250 nM for miR-142-3p) in hybridization mix (600 mM NaCl, 10 mM HEPES, 1 mM EDTA, 5X Denhardts, 50% formamide) for 1 h at 58 °C. Sections were washed with 2X saline-sodium citrate buffer (SSC) for 2 minutes, 0.5X for 2 minutes, 0.2X for 1 minute (in agitation). After washing with sterile PBS, sections were blocked for 15 minutes with 1% BSA, 0.02% Tween 20 and 1% normal goat serum. Hybridization was detected with sheep alkaline phosphatase (AP)-labelled anti-DIG antibody (1:1,500, Roche Applied Science, Basel, Switzerland). Nitro-blue tetrazolium chloride (NBT)/ 5-bromo-4-chloro-3'-indolyphosphate p-toluidine salt (BCIP) was used as chromogenic substrate for AP (1:50 diluted in NTM-T buffer: 100 mM Tris, pH 9.5; 100 mM NaCl; 50 mM MgCl2; 0.05% Tween 20). Negative control assays were performed without probes (sections were blank).
ISH with immunohistochemistry 
For in situ hybridization with double labelling, sections were first incubated in 0.3% H2O2/methanol solution for 20 min to block endogenous peroxidase activity, followed by in situ hybridization and then by immunohistochemistry. Slides were washed with PBS and incubated for 1 h at room temperature with the following primary antibodies prepared in normal antibody diluent (Klinipath, Olen, Belgium): mouse anti-GFAP (1:4,000, Sigma-Aldrich, St. Louis, MO, USA), mouse anti-NeuN (1:2,000, MAB377, Chemicon, Temecula, CA, USA), rabbit anti-Iba1 (1:2,000, Wako Chemicals, Neuss, Germany), mouse anti-HLA-DR/DP/DQ (1:100, clone CR3/43 Agilent, Santa Clara, CA, USA), rabbit anti-TMEM119 (1:500, #HPA051870, Sigma-Aldrich, St. Louis, MO, USA), mouse anti-CD68 (1:200, clone KP1, Dako, Glostrup, Denmark), or rabbit anti-CD8 (1:200, #7103, Dako, Glostrup, Denmark). After washing with PBS, sections were stained with a polymer-based horseradish peroxidase (HRP) immunohistochemistry detection kit (Brightvision plus kit, ImmunoLogic, Duiven, the Netherlands) according to the manufacturer's instructions. The visualization of the antibody-antigen binding was done using 3-amino-9-ethylcarbazole (AEC; Sigma-Aldrich, St. Louis, MO, USA), which in the presence of hydrogen peroxide undergoes chromogenic oxidation catalysed by HRP with the formation of a red precipitate.
Statistical analysis
Statistical analyses were performed using Graphpad prism 5. The Mann-Whitney U-test or Kruskal-Wallis non-parametric test with Dunn's post-hoc test were used for comparisons between groups. A value of p<0.05 was assumed to indicate significant difference.   
